# Unlocking Survival Mechanisms for Metal and Oxidative Stress in the Extremely Acidophilic, Halotolerant *Acidihalobacter* Genus

**DOI:** 10.3390/genes11121392

**Published:** 2020-11-24

**Authors:** Himel Nahreen Khaleque, Homayoun Fathollazadeh, Carolina González, Raihan Shafique, Anna H. Kaksonen, David S. Holmes, Elizabeth L.J. Watkin

**Affiliations:** 1 School of Pharmacy and Biomedical Sciences, Curtin University, Perth 6845, Australia; himelnahreen.khaleque@csiro.au (H.N.K.); homayoun.fathollahzadeh@curtin.edu.au (H.F.); raihan.shafique@curtin.edu.au (R.S.); 2 CSIRO Land and Water, Floreat 6014, Australia; anna.kaksonen@csiro.au; 3 Center for Bioinformatics and Genome Biology, Fundacion Ciencia y Vida, Santiago 7750000, Chile; carola.mgr@gmail.com (C.G.); dsholmes2000@yahoo.com (D.S.H.); 4 Centro de Genómica y Bioinformática, Facultad de Ciencias, Universidad Mayor, Santiago 8580000, Chile; 5 Universidad San Sebastian, Santiago 8320000, Chile

**Keywords:** CopA, Rbr, oxidative stress, metal stress, halotolerant acidophile, bioleaching, horizontal gene transfer

## Abstract

Microorganisms used for the biohydrometallurgical extraction of metals from minerals must be able to survive high levels of metal and oxidative stress found in bioleaching environments. The *Acidihalobacter* genus consists of four species of halotolerant, iron–sulfur-oxidizing acidophiles that are unique in their ability to tolerate chloride and acid stress while simultaneously bioleaching minerals. This paper uses bioinformatic tools to predict the genes and mechanisms used by *Acidihalobacter* members in their defense against a wide range of metals and oxidative stress. Analysis revealed the presence of multiple conserved mechanisms of metal tolerance. *Ac. yilgarnensis* F5^T^, the only member of this genus that oxidizes the mineral chalcopyrite, contained a 39.9 Kb gene cluster consisting of 40 genes encoding mobile elements and an array of proteins with direct functions in copper resistance. The analysis also revealed multiple strategies that the *Acidihalobacter* members can use to tolerate high levels of oxidative stress. Three of the *Acidihalobacter* genomes were found to contain genes encoding catalases, which are not common to acidophilic microorganisms. Of particular interest was a rubrerythrin genomic cluster containing genes that have a polyphyletic origin of stress-related functions.

## 1. Introduction

The *Acidihalobacter* (*Ac*.) genus belongs to the family *Ectothiorhodospiraceae*, order Chromatiales, class Gammaproteobacteria, and phylum Proteobacteria. The genus has four species, which have been validly described as *Ac. ferrooxydans* DSM 14175^T^, *Ac. prosperus* DSM 5130^T^, *Ac. aeolianus* DSM 14174^T^ and *Ac. yilgarnensis* F5^T^ [1,2,3]. The three first strains were isolated from the Vulcano Region, Italy, whereas *Ac. yilgarnensis* F5^T^ was isolated from an acidic, saline drain in the Yilgarn Region of Western Australia. All four species are aerobic, acidophilic and halotolerant, chemolithoautotrophic, and iron- and sulfur-oxidizers [1,2,3]. A unique feature of *Ac. yilgarnensis* F5^T^ is its ability to leach copper from chalcopyrite under saline conditions (18 g/l chloride) [3,4]. The *Acidihalobacter* genus represents a novel group of microorganisms that survive in environments where other acidophiles fail to thrive. This is due to their ability to tolerate high levels of chloride ion simultaneously with acid stress while also bioleaching minerals [4,5,6].

Metals play an essential role in microorganisms as catalysts, enzyme co-factors, protein stabilizers, and electron donors or acceptors in redox processes [7,8]. However, bioleaching environments typically have elevated metal concentrations that can become toxic due to the accumulation of metals in cells. The toxicity effects of metals can result from the formation of coordinate bonds with anions causing blocking of functional groups of enzymes, inhibition of transport systems, displacement of essential metals from their binding sites, and disruption of cell membrane integrity [7,9]. For example, cadmium, nickel, and mercury induce the depletion of glutathione and bind to sulfhydryl groups of proteins. Arsenic has high affinity to sulfhydryl groups of proteins and may react with thiol groups of active sites in enzymes and on thioredoxin, glutathione, and glutaredoxin, and impact protein folding, intracellular redox homeostasis, and detoxification of xenobiotics [10]. The toxicity of metals varies widely depending on microbial species and strains and their strategies for exhibiting metal resistance [11]. Metal tolerance can be conveyed through the efflux of toxic metals from the cells; intra- or extracellular sequestration; enzymatic conversion of metals; exclusion of metals by a permeability barrier; and reduction in sensitivity of cellular targets [7].

Aerobic respiration of microorganisms results in the production of partially reduced reactive oxygen species (ROS), such as hydrogen peroxide (H_2_O_2_), superoxide (O_2_•^−^), and highly reactive hydroxyl radicals (OH•) [10,12,13]. ROS can also be generated through the exposure to environmental factors, such as light, oxidative chemical agents, metals, and minerals [10,14]. It has been shown that an increase in chloride stress also induces an increase of ROS in *Leptospirillum ferriphilum* DSM 14647 [8]. In extremely acidic bioleaching environments, ROS are generated spontaneously on the surfaces of minerals, such as pyrite [15,16,17]. Moreover, redox active metals, such as iron, copper, cobalt, and chromium undergo redox cycling reactions and may induce the generation of ROS [10]. Examples of metal-coupled ROS generating reactions are the Fenton (Reaction (1)) and Haber–Weiss reactions (Reactions (2) and (1), resulting in net reaction 3) ([10,18,19].

H_2_O_2_ + Fe^2+^ → Fe^3+^ + OH^−^ + OH• (1)

O_2_•^−^ + Fe^3+^ → O_2_ + Fe^2+^ (2)

O_2_•^−^ + H_2_O_2_ → O_2_ + OH^−^ + OH• (3)

The ROS can cause mutations to nucleic acids, inactivate proteins, oxidize lipids, and damage other macromolecules and thus decrease cell growth and survival [19,20,21]. The defense mechanisms against ROS include enzymatic transformation, quenching and/or consumption of radicals to prevent ROS accumulation, repair systems for damaged macromolecules, and regulatory loops to control the expression of various response stages. The imbalance between the generation of ROS and their suppression by antioxidant defense mechanisms causes oxidative stress to cells [19]. For example, ROS promotes disulfide bond formation in proteins. Thioredoxin fold proteins (TFPs) are thought to protect damaged proteins from inactivation. Examples of TFPs include thioredoxin, peroxiredoxin, oxidoreductase, glutaredoxin, glutathione S-transferase, and glutathione peroxidase [22].

Previous proteomic studies and comparative genomics have identified mechanisms for chloride tolerance for the members of the *Acidihalobacter* genus; however, high levels of metal and oxidative stress also represent key factors that can compromise the feasibility of bioleaching of metals from minerals [23,24]. There is a limited understanding of the molecular components involved in defense mechanisms in acidophiles in general and in the recently reclassified species of the *Acidihalobacter* genus in particular. Furthermore, acidic brine environments, such as those found in the Yilgarn craton of Western Australia and in the Altiplano region of Northern Chile, have been suggested to be important terrestrial analogs for some Martian environments [25,26,27,28]. Therefore, uncovering the conserved mechanisms as well as evolutionary adaptations of the members of the *Acidihalobacter* genus can inform their effect on the bioleaching of minerals, assist in the identification of novel enzymes for biotechnological processes, and extend the existing theories about the boundaries of life on both Earth and in the universe [5]. 

## 2. Materials and Methods

### Genome Annotation and Comparisons

The genome sequences of *Ac. prosperus* DSM 5130^T^ (JQSG02000000) *Ac. aeolianus* DSM 14174^T^ (CP017448.1), *Ac. ferrooxydans* DSM 14175^T^ (CP019434.1), and *Ac. yilgarnensis* F5^T^ (CP017415.1) were downloaded from the NCBI ftp site (ftp://ftp.ncbi.nlm.nih.gov/). For the purpose of this comparison, the genomes were annotated using the Rapid Annotation using Subsystem Technology (RAST) server (http://rast.nmpdr.org/) using the ClassicRAST annotation scheme [29]. Comparisons were performed using the SEED and RAST servers, Geneious v.10.2.6 bioinformatic software, and an in-house Python pipeline. Metabolic pathways were predicted with Kyoto Encyclopedia of Genes and Genomes (KEGG) (http://www.genome.jp/kegg/). 

Mobile genetic elements (MGEs) as insertion elements, transposes, and truncated transposases in *Acidihalobacter* genomes were predicted with TnpPred [30]. The genome contexts of interest including metal resistance genes, hypothetical genes, and predicted mobile elements were analyzed using STRING [31], Artemis [32], and MAUVE [33]. The genome contexts were manually curated with Artemis [32] and MAUVE [33]. Synteny blocks and conservation of genetic element contexts in *Acidihalobacter* genomes were determined by MAUVE [33]. 

Genes and mechanisms involved in metal and oxidative stress resistance were identified through a literature search. Identification of similar genes in *Acidihalobacter* genomes was made with BLASTp and BLASTx [34] comparison using a minimal *E*-value cutoff of 1e-5, followed by a manual curation to confirm the presence of domains of interest, discarding sequences with different motifs to analyzed and truncated domains. Selected sequences were used for alignments with the MAFFT alignment tool [35,36]. Conservation of sequences domains and visualization was made with Jalview [37], WebLogo [38,39], and Aliview [40]

The *cop*A metal-binding motifs including CXXC, HXXH, CXC, and HXH [41,42] were searched in the alignment of *Acidihalobacter* genes with *cop*A domain. The metal-binding motifs for the copper translocating type ATPases (CXXC and CXC) identified are highlighted and represented in Figure S1.

## 3. Results and Discussion

Features of the genomes of the *Acidihalobacter* genus members have been described previously. In this study, further bioinformatic analyses predicted multiple genes and pathways involved in tolerance and resistance to high levels of metal and oxidative stress, which are discussed in detail below.

### 3.1. Strategies to Cope with High Metal and Metalloid Concentrations

As mentioned previously, bioleaching environments are rich in metals that can be toxic to microorganisms when they accumulate inside cells. The toxicity of metals and the mechanisms for metal resistance in bioleaching microorganisms vary widely between microbial species and strains [11]. Genome analysis revealed multiple genes and pathways that *Acidihalobacter* species may use to tolerate heavy metal and metalloid stresses. 

#### 3.1.1. Copper

Copper is an essential trace element for all aerobic organisms and functions as a cofactor in enzymes responsible for a wide variety of redox reactions. However, if copper homeostasis is not maintained, its redox properties can result in toxicity. Chalcopyrite (CuFe_2_S_2_) is the most abundant copper-containing mineral in the lithosphere and, unlike many other minerals, is recalcitrant to industrial hydrometallurgical recovery of copper (bioleaching). It has been shown by Falagán and Johnson [43] that chloride acts as a ligand for copper ions and results in the formation of both positively and negatively charged complexes that at low pH can be more toxic to cells than copper (II) or chloride ions alone. Therefore, the discovery and characterization of bacteria like *Ac. yilgarnensis* F5^T^ [6] that can tolerate low pH and high concentrations of chloride ion, while oxidizing chalcopyrite to release copper, is of major benefit to the biomining industry. 

Several open reading frames (ORFs) have been proposed to code for putative proteins related to copper resistance in the genomes of the *Acidihalobacter* species, among which genes for the copper translocating P-type ATPase (copA) have been identified to have an important role [44]. It was recently noted by Li and Zhu [41] that two different types of *cop*A genes exist which encode proteins with different substrate affinity. The P-type CopA ATPase uses ATP and binds Cu(I) for transport. It contains the N-terminal CXXC (or HXXH as a variant) motif and has CXC as its transmembrane metal-binding motif. However, the multicopper CopA has an HXH as its motif and uses histidine as its metal-binding residue. Therefore, to determine the type of CopA proteins used by the members of the *Acidihalobacter* genus, an in-depth analysis was undertaken. The majority of CopA proteins in all *Acidihalobacter* members was the P-type ATPase type (Figure S1). The genes where no motifs were found (*Ac. ferrooxydans DSM 14175^T^*peg. 1233, *Ac. aeolianus DSM 14174^T^* peg.1366, *Ac. yilgarnensis* F5^T^ peg. 1599, *Ac. prosperus* DSM 5130^T^ peg.679 and peg.2044) were identified as multicopper oxidases. Multicopper oxidases have previously been described to have roles in the copper resistance and tolerance in other bacteria [45,46,47]. They are responsible for copper detoxification through the use of a variety of substrates such as phenolic compounds or siderophores, by catalyzing the single-electron oxidation of four substrate equivalents coupled with the reduction of oxygen to water [48,49].

Further genome analyses revealed the presence of copper-resistance proteins, CopB, CopC, and CopD, which are known to assist in the uptake and transport of copper to the cytoplasm for subsequent sequestration or expulsion [28]. *Ac. prosperus* DSM 5130^T^, *Ac. aeolianus* DSM 14174^T^, and *Ac. yilgarnensis* F5^T^ all have the *cop*C and *cop*D copper uptake genes as well as the *cop*B gene, whereas these genes are absent in *Ac. ferrooxydans* DSM 14175^T^. Furthermore, *cus*A, which codes for a cation efflux pump, is also known to be involved in copper resistance in the salt-sensitive acidophile, *Acidithiobacillus* (A.) *ferrooxidans.* The gene for CusA was absent in *Ac. ferrooxydans DSM 14175^T^*, but present in *Ac. yilgarnensis* F5^T^ (6 copies), *Ac. aeolianus* DSM 14174^T^ (5 copies), and *Ac. prosperus* DSM 5130^T^ (3 copies). 

**Figure 1:**
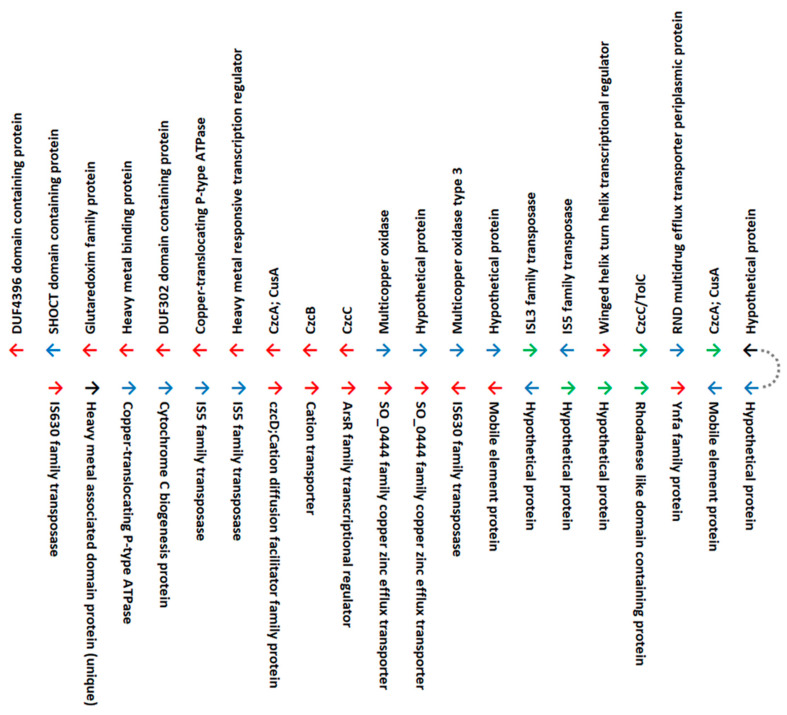
Region of the *Acidihalobacter yilgarnensis* F5^T^ identified to be rich in mobile genetic elements and copper-resistance genes. Red arrows = genes with similarity to orthologs in acidophiles; blue arrows = genes with similarity to orthologs in neutrophilic, halophilic, and halotolerant organisms or to other non-acidophiles, green arrows = genes with similarity to orthologs in halotolerant microorganisms, black arrows = genes unique to *Acidihalobacter*. Dotted line represents contiguity. Additional information can be found in Supplementary File 1, Table S1.

A cluster of genes found only in *Ac. yilgarnensis* F5^T^ contains multiple genes (~40) encoding heavy metal-resistance transporters, metal-binding proteins, transcriptional regulators, and efflux proteins (Figure 1). The cluster is flanked by mobile elements, including insertion sequences IS5, IS630, ISL3 families, and DUF4396 and DUF302 domain-containing proteins. An analysis of the best BLASTx hits (Table S1) of these genes suggests that most are derived from horizontal gene transfer from acidophilic bacteria, while others may have entered by vertical descent from halophilic or halotolerant common ancestors. Upon comparison with the copper tolerance genes in the other species it was observed that the *cop*A genes were located in various regions of the genome, but were not organized as a gene cluster with other heavy metal tolerance genes. *Ac. yilgarnensis* F5^T^ is the only pure mesophilic, aerobic, iron-, and sulfur-oxidizing isolate of the *Acidihalobacter* genus which has been shown to bioleach chalcopyrite at 18 g/l chloride ion. Here, we hypothesize that the ability of *Ac. yilgarnensis* F5^T^ to successfully bioleach chalcopyrite is due to the presence of the cluster of genes encoding additional proteins with roles in metal resistance.

#### 3.1.2. Other Divalent Heavy Metals and Metalloids

Mercury (Hg) is amongst the most toxic heavy metals due to its high affinity binding to sulfhydryl ligands in amino acids, thereby resulting in changes in protein structure that may lead to a loss of function [50]. Mercuric reductase (MerA) is a homodimeric flavin-dependent disulfide oxidoreductase that has been identified as the central enzyme in the microbial mercury resistance system as it catalyzes the reduction of Hg(II) to volatile Hg(0) [51]. In addition to MerA, *mer* operons often encode for proteins involved in regulation (MerR, MerD), Hg scavenging/binding (MerP), and organomercury degradation (MerB), as well as one or more inner membrane scanning transport proteins (MerT, MerC, MerE, MerF, MerG) [51]. The genomes of *Acidihalobacter* showed differences in the organization of their *mer* genes. *Ac. prosperus* DSM 5130^T^ and *Ac. yilgarnensis* F5^T^ contained a copy of *mer*A, *mer*P, and *mer*T as part of one cluster. However, in *Ac. aeolianus* DSM 14174^T^, the *mer*P was replaced by a cation transporter. This cluster was not present in *Ac. ferrooxydans* DSM 14175^T^. Additional *mer* gene clusters containing *mer*C, *mer*R, and *mer*A were identified on separate locations of the genomes in *Ac. prosperus* DSM 5130^T^, *Ac. ferrooxydans* DSM 14175^T^, and *Ac. yilgarnensis* F5^T^, but not in *Ac. aeolianus* DSM 14174^T.^

Arsenic is a highly toxic metalloid that exists in the environment mainly in inorganic forms, such as trivalent arsenite (As(III)) and pentavalent arsenate (As(V)). To survive in arsenic-rich environments, organisms have developed multiple metabolic strategies for its detoxification. A broad diversity of arsenic resistance system (*ars*) operons has been characterized in different species (14–18). However, the most extensively studied for arsenic detoxification are those that include the genes *ars*RBC or *ars*RDABC. The gene products are responsible for reducing cytoplasmic toxic As(V) to less toxic As(III) by (i) the uptake of arsenate by phosphate transporters and uptake of arsenite by aquaglyceroporins, (ii) transformation of As(V) to As(III) by arsenate reductases, and (iii) extrusion of As(III) by arsenite efflux permeases (9). Due to the presence of multiple arsenate reductase genes in the *Acidihalobacter* genomes, it is speculated that arsenic detoxification in this genus occurs through the aforementioned *ars* system. Proteomic studies performed on *Ac. aeolianus* DSM 14174^T^ have shown the increase in abundance of arsenic reductase in this strain in the presence of high salt concentrations, suggesting increased detoxification when the isolate is under stress [52]. The genome of *Ac. yilgarnensis* F5^T^ contained 3 copies of the arsenate reductase genes; however, the remaining *Acidihalobacter* genomes contained 4 copies of this gene. However, in each isolate, only one of these arsenate reductase genes was present as part of the *ars*RBC operons consisting of an *ars*R family transcriptional regulator (repressor), *ars*B arsenic transporter/efflux pump, and *ars*C arsenate reductase. In *Ac. aeolianus* DSM 14174^T^ and *Ac. ferrooxydans* DSM 14175^T^, one of the arsenate reductases was directly upstream of a *wrb*A flavoprotein with known roles in oxidative stress. In *Ac. ferrooxydans* DSM 14175^T^, one arsenate reductase gene was downstream of the *ars*R transcriptional regulator as well as a cadmium-induced protein. 

Apart from the aforementioned tolerance genes, multiple genes for putative cation/multidrug or heavy metal efflux pumps (zinc, cadmium, lead, cobalt) and transporting ATPases were detected in all the genomes of the *Acidihalobacter* species, which are possibly used to export heavy metals across the membranes, as has been described previously by Dopson and Holmes [53]. Amongst these, the *czc*CBA has been found to be essential in the expression of cobalt, zinc, cadmium, and nickel resistance [50]. Nickel transport and metabolism occurs through the high-affinity nickel transport HoxN, hydrogenase nickel incorporation protein HupN, and high-affinity nickel-transport NixA [54,55,56]. Multiple copies of transporter encoding genes were found in all *Acidihalobacter* genomes. Further study of these translocating ATPases and transporters is required to determine their specificity to different metals, but it is hypothesized that these all play an important role in the expulsion of heavy metals out of the cells.

#### 3.1.3. The poly-P Mechanism

**Figure 2:**
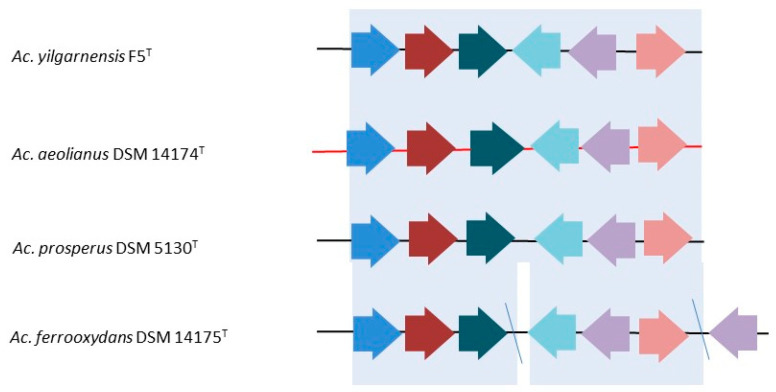
Genetic organization of the *ppx* and *ppk* genes in the four species of *Acidihalobacter*. Blue arrows = phosphate regulon transcriptional regulatory protein (PhoB); brown arrows = phosphate regulon sensor protein (PhoR); green arrows = exopolyphosphatase (EC 3.6.1.11) (Ppx); light blue arrows = lipoprotein, putative (Lipo); light purple arrows = polyphosphate kinase (Ppk); pink arrows = phosphate transport system regulatory protein (PhoU). Blue lines indicate that the genes are not contiguous. Red line indicates reverse orientation.

Metal ion removal in microorganisms may also be achieved through other mechanisms such as the complexation of metals with sulfate ions and the competition of metal ions and protons at low pH [53,57,58,59,60]. An additional mechanism for heavy metal tolerance in the *Acidihalobacter* species may be through the poly-P dependent mechanism, as reviewed by Navarro and von Bernath [44]. In this mechanism, acidophiles such as *Acidithiobacillus ferrooxidans*, *A. caldus*, *A. thiooxidans*, and the archaea *Sulfolobus metallicus* can synthesize and accumulate long polymers of inorganic polyphosphates (poly-P) from ATP using the enzyme polyphosphate kinase (Ppk) [61,62,63,64,65]. In the presence of copper, the polyphosphatase (Ppx) enzyme that breaks down poly-P is activated and this causes the release of inorganic phosphate [66]. The inorganic phosphate can then bind to metal cations in the cytoplasm and be excreted to the periplasmic space through inorganic phosphate carriers [44,67]. It has previously been suggested that the presence of Ppk and Ppx on the same operon can result in the co-transcription of these genes, thereby limiting the accumulation of polyphosphate [68]. This could help to rapidly release inorganic phosphate from polyphosphates for metal removal when the cells are faced with high metal stress. However, in the salt-sensitive *A. ferrooxidans*, it was found that the genes for Ppk and Ppx were not colocalized as a gene cluster, suggesting that the genes allow for accumulation of polyphosphate in this bacterium [68]. In the *Acidihalobacter* species, the genes for Ppx and Ppk were found to form part of a single gene cluster, except in *Ac. ferrooxydans* DSM 14175^T^ where the genes were found in different locations of the genomes (Figure 2). Furthermore, *Ac. ferrooxydans* DSM 14175^T^ contained a polyphosphate kinase 2 gene that was not present in the genomes of the other *Acidihalobacter* members. This suggests that, like *A. ferrooxidans*, *Ac. ferrooxydans* DSM 14175^T^ is more likely to accumulate polyphosphates rather than breaking them down to release inorganic phosphates as a strategy for metal resistance as in the other *Acidihalobacter* members.

### 3.2. Strategies to Tolerate Oxidative Stress

The need for bioleaching microorganisms to maintain high oxidation rates for cellular metabolism requires them to have systems in play to protect against the direct or indirect oxidative damage caused to their DNA, proteins, and membranes by ROS species such as hydrogen peroxide, super oxide radicals, and organic peroxides [19,69]. As previously mentioned, both the presence of high concentrations of redox-active metals as well as chloride stress may be responsible for the generation of ROS in the *Acidihalobacter* species that survive in saline bioleaching environments [70]. Therefore, the genomes of the *Acidihalobacter* isolates were searched for predicted genes and pathways potentially involved in protection of the cells from ROS. 

#### 3.2.1. Rubrerythrin and Neighborhood Genes 

Rbr is a member of the ferritin-like superfamily [71] and has been implicated in stress survival in many bacteria [72] and archaea [73]. It has been experimentally verified to function as a scavenger of ROS using a di-iron center to reduce H_2_O_2_ and organic hyperperoxide to water [74,75,76,77,78].

**Figure 3:**
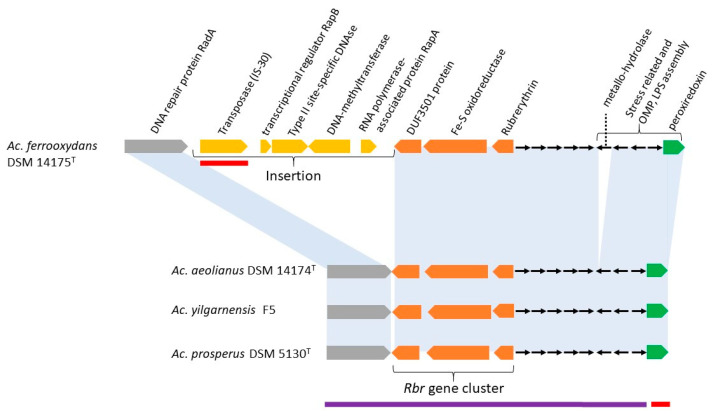
Genomic context of rubrerythrin (*rbr*) and neighboring genes in the four species of *Acidihalobacter*. Red underlining = genes with similarity to orthologs in acidophiles; purple underlining = genes with similarity to orthologs in neutrophilic halophilic and halotolerant organisms or to other non-acidophiles (Supplementary File 2).

Rbr is found in all four species of *Acidihalobacter* and is located in a conserved three gene cluster that includes genes potentially encoding DUF 3501 (a protein of unknown function) and a Fe-S oxidoreductase (Figure 3). These genes have been proposed to have evolved as auxiliary functions that promote the activity of Rbr in aerobic conditions [72] (Figure 3). This association is not unexpected since *Acidihalobacter* are known to be aerobes [1]. The predicted domain structure of the *Acidihalobacter* Rbr shows that it has an RFO (rubrerythrin-associated Fe-S oxidoreductase) domain (Supplementary File 2).

In proximity to the *rbr* gene cluster is *prx*, predicted to encode peroxiredoxin of the Bcp-Q family, where Bcp = bacterioferritin comigratory protein [79] (Figure 3). The role of peroxiredoxins in the detoxification and removal of organic peroxides has also been described in other acidophiles, such as *Sulfolobus solfataricus*, *Acidithiomicrobium* spp., *Alicyclobacillus* spp., and *Sulfobacillus* spp. [19,80,81]. PrxBcp-Q has high peroxidase activity and broad substrate specificity, reducing peroxides such as H_2_O_2_, lipid peroxide, and peroxynitrite [82,83,84,85,86,87]. Nearby are genes involved in lipid and outer membrane protein formation, so it is possible that *prx* Bcp-Q is involved in a stress response to membrane components in *Acidihalobacter*. An alternative hypothesis is that it is involved in the reduction of the radical peroxynitrite. In low pH conditions, such as are found in environments inhabited by *Acidihalobacter*, exogenous nitrite is rapidly converted to nitrous acid which decomposes to various nitrogen oxides including nitric oxide and peroxynitrite that can diffuse through the membrane [88,89]. It is interesting to note that *Acidihalobacter prx*Bcp-Q has significant sequence similarity to *prx*Bcp-Q of moderate acidophiles belonging to the *Burkholderiaceae* which may reflect a function in acidic stress responses (Supplementary File 2). Nearby are several genes with potential functions in stress and OMP/lipid assembly. There is also a gene predicted to encode an metallo β-lactamase (MBL) fold metallo-hydrolase that is found only in *Ac. ferrooxydans* DSM 14175^T^ (Figure 3). MBL fold metallo-hydrolases have diverse functions including important roles in antibiotic resistance [90]. They have also been shown to be involved in stress responses to metalloids in acidic environments and play a crucial role in tolerance to high-arsenic sulfide ore concentrates and arsenic, in particular [91]. Some are also highly mobile between species [92]. These properties could explain their presence in *Ac. ferrooxydans* DSM 14175^T^. Immediately downstream of the *Rbr* cluster is a group of genes found only in *Ac. ferrooxydans* DSM 14175^T^ (Figure 3). One of these genes is predicted to be a site-specific insertion sequence of the IS30 family with canonical DDE and DNA binding helix-turn-helix motifs (Supplementary File 2). It has strong sequence similarity to IS30 in multiple species of the extremely acidophilic *Acidithiobacillus* genus. Strikingly, it is more similar to IS30 sequences of the *Acidithiobacillus* genus than to ones in the family Ectothiorhodospiraceae, to which the *Acidihalobacter* genus belongs. This provides evidence for the transference by horizontal gene transfer (HGT) of IS30, and possibly also the other contiguous genes that appear in the unique insertion in *Ac. ferrooxydans* DSM 14175^T^ HGT shown in Figure 3. IS30 has been shown in other organisms to be involved in environmental adaption and modification of the expression of neighboring genes at the integration site [93,94], including adaptive response to oxidative stress [95]. 

The contiguous genes associated with IS30 potentially encode the following proteins: (i) A transcriptional regulator of the XRE family with weak similarity to the toxin-antitoxin HipB; (ii) a site specific nuclease type II (Sma-like); (iii) a hypothetical protein with similarity to a site-specific DNA-methyltransferase; (iv) a truncated hypothetical protein with similarity to the toxin-antitoxin HipA that may function with the nearby HipB to form a toxin–antitoxin module potentially involved in the production of non-growing “persister” cells that can lie dormant during stress conditions [96], including metal and oxidative stress and perhaps other stresses. The toxin–antitoxin module has also been implicated in contact inhibition of cell growth, allowing bacteria to recognize kin cells in mixed bacterial populations [97]. Recently, it was shown that the toxin–antitoxin module can also affect membrane composition [98]. However, as a caveat, it is not known whether the truncated form of HipAB in *Ac. ferrooxydans* DSM 14175^T^ is functional in any of the above roles. The site-specific nuclease type II is most similar to the restriction enzyme Sma whose DNA recognition site is an inverted repeat of six base pairs creating blunt ends on cutting that can be ligated to other blunt-ended DNA. Its activity can be inhibited by methylation of the recognition site. It is possible that the adjacent site-specific methyltransferase carries out this function. Thus, when the methylase is functioning, it protects the *Ac. ferrooxydans* DNA from self-damage but could potentially destroy invading non-methylated DNA such as phage and plasmids.

An analysis of the best BLASTp hits of the contiguous genes in the *rbr* region suggests that some are most likely derived by vertical descent from non-acidophilic, halophilic, or halotolerant bacteria, while others may have entered the genome of a common ancestor by HGT, suggesting a polyphyletic origin of stress-related functions (Figure 3).

#### 3.2.2. Catalase and Alkyl Hydroperoxide Reductase

Catalase, along with superoxide dismutase and peroxidase are three of best characterized ROS defense mechanisms across domain bacteria [99]; however, comparative genomics of the oxidative stress response in forty-four bioleaching microorganisms revealed that most acidophiles lack genes encoding classical ROS consumption enzymes, particularly catalase [19]. Furthermore, multi-omics of *L. ferriphilum* DSM 14647 showed that homologs of catalases are absent [100]. Unexpectedly, in this study, genes encoding catalase were identified in the genomes of *Ac. yilgarnensis* F5^T^, *Ac. prosperus* DSM 5130^T^, and *Ac. ferrooxydans* DSM 14175^T^. However, homologs of the catalase genes were not found in the genome of *Ac. aeolianus* DSM 14174^T^. Catalases are potentially more efficient at scavenging H_2_O_2_ at high concentrations [69]; therefore, their presence in *Ac. yilgarnensis* F5^T^*, Ac. prosperus* DSM 5130^T^*,* and *Ac. ferrooxydans* DSM 14175^T^ suggests the presence of an alternative oxidative stress mitigation strategy to promote their survival under oxidative challenges in bioleaching environments.

Upon further analysis through BLASTx, it was revealed that the sequences of the catalase genes for *Ac. yilgarnensis* F5^T^ and *Ac. prosperus* DSM 5130^T^ were homologous (77% protein sequence identity) to those from a novel sulfur oxidizing bacterium isolated from lake sediment, *Sulfuricaulis limicola* [101] (Table S2). However, the catalase gene for *Ac. ferrooxydans* DSM 14175^T^ was not found to be homologous to those in the other *Acidihalobacter* genomes but was rather found to share 77% protein sequence similarity to catalase genes from uncultured bacteria from biostimulated petroleum-contaminated soil [102] or catalase genes from halotolerant *Nitratireductor* species [103,104]. Therefore, it is likely that these genes were gained from vertical transfer from a halophilic or halotolerant ancestor.

Among other genes associated with oxidative stress management during intercellular and extracellular acidification, alkyl hydroperoxide reductase subunit C (AhpC) is widely conserved in all the *Acidihalobacter* species. Under low stress conditions, AhpC catalyzes the reduction of endogenously generated hydrogen peroxide and organic hydroperoxides to water and alcohols [69,105]. The genomes of the strains *Ac. yilgarnensis* F5^T^ and *Ac. ferrooxydans* DSM 14175^T^ each have two copies of *ahpC*, whereas genomes of *Ac. aeolianus* DSM 14174^T^ and *Ac. prosperus* DSM 5130^T^ have one copy of *ahpC*. It has been shown that the mutual regulation and expression of catalase and *ahpC* genes results in dual response to oxidative stress [106]. This is consistent with previous studies that suggested proteobacteria have the strongest ROS defense signature across domain bacteria [23,99]. It has also been suggested that during high ROS stress in bacteria, the hydrogen peroxide-inducible genes activator protein regulator (OxyR) is activated and an increased expression and activity of the oxidative stress responses of AhpC and catalase are observed [23,107]. Genes encoding OxyR were identified in all of the *Acidihalobacter* species. It is perhaps more interesting to note that all of the *Acidihalobacter* species also contain genes encoding putative *Alkanesulfonate monooxygenase* (*ssuD*). It is likely that under sulfate starvation the ability of the SsuD to utilize alkanesulfonate was linked to oxidative stress response, with upregulation of *ahpC* and catalases to counter oxidative stress [108,109]. 

#### 3.2.3. Other Mechanisms of Oxidative Stress Tolerance

Cell survival is largely dependent on the maintenance of disulfide states through the control of thiol/disulfide redox balance in the cytoplasm by either the thioredoxin reductase/thioredoxin system or the glutathione reductase/glutathione/glutaredoxin pathways. Both these protein systems play a large role in the defense against oxidative stress.

The thioredoxin system consists of thioredoxin reductase (TR), which regulates the intracellular redox environment through the reduction of thioredoxin (Trx) in an NADH-dependent fashion [110]. The system is involved in the regeneration of oxidative damaged proteins. It also protects against oxygen damage by modulating the activity of redox stressors and in the donation of hydrogen to detoxification enzymes that are key to the oxidative stress response [111,112]. Many studies have been performed on both aerobic and anaerobic TR/Trx systems, confirming their roles in defense against oxidative damage in both life forms and also extending the properties to reveal different types of electron donors in the different forms [113,114,115,116,117,118,119]. The acidophilic bacterium, *L. ferriphilum* DSM 14647, which can tolerate high concentrations of iron, salt, and other redox active metals, was studied for its response to oxidative stress and found to increase transcriptional activation of the genes encoding Trx and the TR enzyme, suggesting that the thioredoxin-based thiol/disulfide system plays an important role in redox protection of this isolate under extreme environmental oxidative conditions [120]. Similarly, it is likely that the *Acidihalobacter* species also use the TR/Trx proteins to address the challenge of oxidative stress as multiple copies of both genes were found in the genomes of all of the strains. 

Genes for thiol peroxidase (Tpx) have been identified in a number of acidophilic microorganisms, including *A. caldus* and *Sulfobacillus acidophilus* [19]. Tpx is a periplasmic hydrogen peroxide scavenger, functioning as a thioredoxin system-dependent protein antioxidant [121]. The genomes of *Acidihalobacter* showed both thioredoxin system protein (Tpx type) as well as a bcp type. It has previously been shown in *Campylobacter jejunii* that the Tpx type uses only hydrogen peroxide as a substrate while the bcp-type also reduces organic peroxides [122]. Therefore, it can be assumed that the *Acidihalobacter* is able to use both types of substrates to help protect against oxidative stress damage.

The glutathione system, consisting of glutaredoxins (Grx), glutathione transferase (GST), glutathione reductase (GR), and NADPH, has been experimentally verified to be another important thiol-disulfide exchange system that represents the main cytoplasmic cellular redox buffer in *Escherichia coli* and other neutrophiles [123]. Glutaredoxins in this system are responsible for catalyzing the glutathionylation and deglutathionylation, while GST catalyzes the reduction of hydroperoxides. GR maintains the ratio of glutathione disulfide and glutathione (GSH) by using NADH to reduce the glutathione disulfide into two molecules of GSH [124,125]. Genes for the glutathione system are present on the genomes of all the *Acidihalobacter* spp. This includes glutathione biosynthesis genes such as glutamate–cysteine ligase, the glutathione synthase, and γ-glutamyltransferase. Furthermore, of the four families of glutaredoxins in the KEGG database—glutaredoxin 1, glutaredoxin 2, glutaredoxin 3, and monothiol glutaredoxin—the genomes of the *Acidihalobacter* species were found to only contain glutaredoxin 3 and the monothiol glutaredoxin but not glutaredoxin 1. However, a glutaredoxin named GR_N was found and upon further analysis was identified as homologous to a glutathione S-transferase N-terminal domain-containing protein, which has potential roles in glutathione binding as well as glutathione transferase activity. Apart from the glutathione synthesis and glutaredoxin genes, there were also enzymes such as glutathione peroxidase on all the genomes of the *Acidihalobacter* species. These may have a role in salvaging damaged proteins and lipids, as has been described for the acidophilic *Ferrovum* JA12 strain [49].

Manganese-iron-type superoxide dismutase enzymes can convert superoxide radicals into hydrogen peroxide [126]. Superoxide dismutase genes are commonly found in the genomes of acidophilic microorganisms, suggesting their ability to transform superoxides (O_2_•–) into hydrogen peroxide (H_2_O_2_) as a detoxification strategy [12,19]. The genome analysis of the four *Acidihalobacter* species showed the presence of manganese-iron-type superoxide dismutases, suggesting they use a similar detoxification strategy in their defense against damaging superoxides.

**Figure 4:**
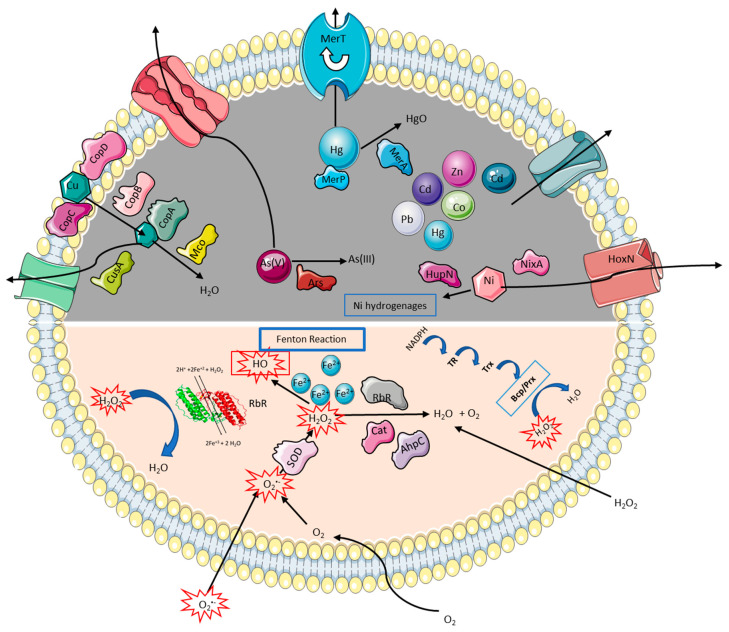
General model of metal (dark gray shading) and oxidative stress (pink shading) tolerance mechanisms used by all members of the *Acidihalobacter* genus. Alkyl hydroperoxide reductase subunit C (AhpC); arsenic resistance protein (Ars); bcp-type peroxiredoxin (BCP/Prx); catalase (Cat); copper translocating P-type ATPase (copA); copper-resistance protein B (CopB); copper-resistance protein C (CopC); copper-resistance protein D (CopD); high-affinity nickel transport protein (HoxN); hydrogenase nickel incorporation protein (HupN); high-affinity nickel-transport protein (NixA); mercuric reductase (MerA); mercury scavenging/binding (MerP); mercury transporting protein (MerT;) multicopper oxidase (Mco); rubrerythrin (Rbr); superoxide dismutase (SOD); thioredoxin (Trx); thioredoxin reductase (TR).

A model of the general responses to metal and oxidative stress for all *Acidihalobacter* species is presented in Figure 4.

## 4. Conclusions

The genomes of the *Acidihalobacter* isolates contained multiple genes that have roles in tolerance to metals and oxidative stress. Some of these genes appear to have been gained through vertical gene transfer from a halophilic or halotolerant ancestor (e.g., catalase and rubrerythrin), whereas others appear to have entered an ancestral genome via horizontal gene transfer from acidophile lineages (e.g., heavy metal resistance genes). A 39.9 Kb DNA segment was found only in the genome of *Ac. yilgarnensis* F5^T^. This segment contains multiple copies of transposes and several copies of copper and other heavy metal associated genes. It is hypothesized that this segment confers properties that promote the bioleaching of the recalcitrant mineral chalcopyrite.

## Supplementary Materials

The following are available online at https://www.mdpi.com/2073-4425/11/12/1392/s1, **Figure S1**: Identification of the metal binding motifs for potential *CopA* proteins in the *Acidihalobacter* genomes compared with copA sequences from members of Firmicutes (*Bacillus subtilis*), CFB group bacteria (*Flavobacterium branchiophilum*), Actinobacteria (*Propionibacterium freudenreichii*), Cyanobacteria (*Synechococcus sp RCC307*), Betaproteobacteria (*Bordetella pertussis*), Deltaproteobacteria (*Geobacter sulfurreducens*), Epsilonproteobacteria (*Helicobacter pylori*), Alphaproteobacteria (*Sphingobium japonicum*) and Gammaproteobacteria (*Escherichia coli*). **Table S1**: BLASTx analysis of the *Acidihalobacter yilgarnensis* F5^T^ operon containing mobile genetic elements and copper resistance genes. **Table S2:** BLASTx analysis of the sequences of the catalase genes for *Ac. yilgarnensis* F5^T^, *Ac. prosperus* DSM 5130^T^ and *Ac. ferrooxydans* DSM 14175^T^. Homologs of the catalase genes were not found in the genome of *Ac. aeolianus* DSM 14174^T^.
