# Supplementary material for: Unlocking Survival Mechanisms for Metal and Oxidative Stress in the Extremely Acidophilic, Halotolerant Acidihalobacter Genus
Source: Genes (Basel). 2020 Nov 24;11(12):1392. doi: 10.3390/genes11121392 (PMC7760498; doi:10.3390/genes11121392)
Supplement: Supplementary file 1 [file genes-11-01392-s001.pdf]

Supplementary File 1.

Unlocking survival mechanisms for metal and oxidative stress in the extremely acidophilic, halotolerant *Acidihalobacter* genus

Himel Nahreen Khaleque[1,5], Homayoun Fathollazadeh[1], Carolina González[2,4], Raihan Shafique[1], Anna H. Kaksonen[5], David S. Holmes[2,3,4] and Elizabeth L.J. Watkin[1*]

[1]School of Pharmacy and Biomedical Sciences, Curtin University, Perth, Australia; [2]Center for Bioinformatics and Genome Biology, Fundacion Ciencia y Vida, Santiago, Chile; [3]Universidad San Sebastian, Santiago Chile; [4]Centro de Genómica y Bioinformática, Facultad de Ciencias, Universidad Mayor, Santiago, Chile; and [5]CSIRO Land and Water, Floreat, Australia

*Correspondence: E.Watkin@curtin.edu.au; Tel.: (+61 8 92662955)

Table S1. BLASTx analysis of the *Acidihalobacter yilgarnensis* F5^T operon containing mobile genetic elements and copper resistance genes. Next best hits in red = genes with similarity to orthologs in acidophiles; green = genes with similarity to orthologs in neutrophilic halophilic and halotolerant organisms or to other non-acidophiles, black = unique to *Acidihalobacter*, blue = genes with similarity to orthologs found across the domain Bacteria.

**peg. No**	**RAST annotation**	**NCBI annotation**	**Ident (%) to *Acidhalocbacter* sp.**	**NCBI accession**	**Next best hit (NBH)**	**Ident (%) to NBH**	**NCBI accession**
2773	FIG00761799: membrane protein CDS	DUF4396 domain-containing protein [Acidihalobacter prosperus]	100%	WP_070080076.1	DUF4396 domain-containing protein [Acidiferrobacter sp. SPIII_3]	55.02%	WP_110138179.1
2774	Hypothetical protein CDS	hypothetical protein BI364_13455 [Acidihalobacter prosperus]	100%	AOU98831.1	SHOCT domain-containing protein [Thermus caldilimi]	80.00%	WP_135257951.1
2775	Hypothetical protein CDS	glutaredoxin family protein [Acidihalobacter prosperus]	100%	WP_070079187.1	glutathione S-transferase N-terminal domain-containing protein [Acidithiobacillus thiooxidans]	77.27%	WP_024895041.1
2776	Heavy metal binding protein	heavy-metal-associated domain-containing protein [Acidihalobacter prosperus]	100%	WP_070079188.1	cation transporter [Acidithiobacillus thiooxidans]	77.27%	WP_031572397.1
2777	hypothetical protein CDS	DUF302 domain-containing protein [Acidithiobacillus ferrooxidans]	75.56%	WP_064219123.1	DUF302 domain-containing protein [Acidithiobacillus thiooxidans]	77.27%	WP_065973816.1

2778	Lead, cadmium, zinc and mercury transporting ATPase (EC 3.6.3.3) (EC 3.6.3.5); Copper-translocating P-type ATPase (EC 3.6.3.4) CDS	copper-translocating P-type ATPase [Acidihalobacter prosperus]	100%	WP_070079189.1	copper-translocating P-type ATPase [Acidithiobacillus ferrivorans]	77.27%	WP_014027734.1
2779	Transcriptional regulator, MerR family CDS	heavy metal-responsive transcriptional regulator [Acidihalobacter prosperus]	100%	WP_070079190.1	heavy metal-responsive transcriptional regulator [Acidithiobacillus sp. SH]	77.27%	WP_101536668.1
2780	Cobalt-zinc-cadmium resistance protein CzcA; Cation efflux system protein CusA CDS	efflux RND transporter permease subunit [Acidihalobacter prosperus]	100%	WP_070079191.1	CusA/CzcA family heavy metal efflux RND transporter [Thiomonas sp. 13-66-29]	86.86%	OZB60967.1
2781	Cobalt/zinc/cadmium efflux RND transporter, membrane fusion protein, CzcB family CDS	efflux RND transporter periplasmic adaptor subunit [Acidihalobacter prosperus]	100%	WP_070079192.1	efflux transporter periplasmic adaptor subunit [Thiomonas sp. 13-66-29]	86.86%	OZB60968.1
2782	Heavy metal RND efflux outer membrane protein, CzcC family CDS	TolC family protein [Acidihalobacter prosperus]	100%	WP_083251402.1	cobalt transporter [Thiomonas sp. 13-66-29]	86.86%	OZB60969.1
2783	Multicopper oxidase CDS	multicopper oxidase domain-containing protein	100.00%	WP_083251403.1	copper oxidase [Candidatus Dadabacteria bacterium]	63.19%	RMG85594.1

		[Acidihalobacter prosperus]					
2784	Hypothetical protein	hypothetical protein [Acidihalobacter prosperus]	100%	WP_070079194.1	TPA: hypothetical protein [Bacteroidetes bacterium]	36.76%	HFB61684.1
2785	Multicopper oxidase type 3	hypothetical protein [Acidihalobacter prosperus]	100%	WP_156782761.1	multicopper oxidase domain-containing protein [Rhodanobacter glycinis]	42.42%	WP_147628282.1
2786	Hypothetical protein	hypothetical protein BI364_13515 [Acidihalobacter prosperus]	100.00%	AOU98841.1	hypothetical protein [Acidihalobacter prosperus] *unique to Acidihalobacter*	?58.46%	WP_145930795.1
2787	Hypothetical protein	ISL3 family transposase [Acidihalobacter prosperus]	100.00%	WP_070079197.1	Transposase [Dokdonella immobilis]	84.86%	SFN71931.1
2788	Mobile element protein	IS5 family transposase [Acidihalobacter prosperus]	100.00%	WP_070079198.1	IS5 family transposase [Pseudomonas aeruginosa]	85.53%	WP_121335850.1
2789	Cadmium efflux system accessory protein CDS	winged helix-turn-helix transcriptional regulator [Acidihalobacter prosperus]	100.00%	WP_070079199.1	winged helix-turn-helix transcriptional regulator [Burkholderia sp. SRS-46]	83.67%	WP_132028862.1
2790	Heavy metal RND efflux outer	TolC family protein [Acidihalobacter prosperus]	100.00%	WP_083251406.1	Heavy metal RND efflux outer membrane protein,	37.75%	AWN14482.1

	membrane protein, CzcC family CDS				CzcC family [Salinisphaera sp. LB1]		
2791	RND-type multidrug efflux pump, membrane permease CDS	hypothetical protein [Acidihalobacter prosperus]	100.00%	WP_070079201.1	efflux RND transporter periplasmic adaptor subunit [Methylobacillus sp.]	44.62%	MPS48876.1
2792	Cobalt-zinc-cadmium resistance protein CzcA; Cation efflux system protein CusA CDS	efflux RND transporter permease subunit [Acidihalobacter prosperus]	100.00%	WP_070079202.1	efflux RND transporter permease subunit [Salinisphaera sp. LB1]	64.42%	WP_109992211.1
2793	Hypothetical protein	No significant similarity found:Short query sequences/Filtering			Unique		
2794	Hypothetical protein	hypothetical protein [Acidihalobacter prosperus]	99.15%	WP_070079203.1	IS3 family transposase [Rhodanobacter sp.]	90.83%	TAL82703.1
2795	Mobile element protein	IS3 family transposase [Acidihalobacter prosperus]	100.00%	WP_070079204.1	IS3 family transposase [Rhodanobacter sp.]	92.90%	TAL82703.1
2796	Protein of unknown function UPF0060 CDS	YnfA family protein [Acidihalobacter prosperus]	100.00%	WP_070080078.1	YnfA family protein [Acidithiobacillus albertensis]	75.56%	WP_075324141.1
2797	Rhodanese-like domain protein CDS	rhodanese-like domain-containing protein [Acidihalobacter prosperus]	100.00%	WP_083251407.1	rhodanese-like domain-containing protein [Halomonas sp. LBP4]	58.82%	WP_110282853.1

2798	Hypothetical protein	hypothetical protein [Acidihalobacter prosperus]	100.00%	WP_083251408.1	hypothetical protein [Ensifer sp. LCM 4579]	50.00%	WP_116288818.1
2799	Hypothetical protein	helix-turn-helix transcriptional regulator [Acidihalobacter prosperus]	100.00%	WP_070079206.1	transcriptional regulator [Halomonas sp. TBZ202]	59.02%	TFH87774.1
2800	Hypothetical protein	hypothetical protein [Acidihalobacter prosperus]	100.00%	WP_070079207.1	hypothetical protein [Aromatoleum toluclasticum]	61.76%	WP_018991669.1
2801	Mobile element protein	IS630 family transposase [Acidihalobacter prosperus]	89.55%	WP_070078097.1	IS630 family transposase [Thiomonas sp. FB-Cd]	82.09%	WP_031406767.1
2802	Mobile element protein	winged helix-turn-helix domain-containing protein [Acidihalobacter prosperus]	100%	WP_083251409.1	IS630 family transposase [Thiomonas sp. FB-Cd]	82.35%	WP_031406767.1
2803	Transporter CDS	SO_0444 family Cu/Zn efflux transporter [Acidihalobacter prosperus]	93.44%	WP_052064744.1	permease [Acidithiobacillus albertensis]	70.13%	WP_075323804.1
2804	Transporter CDS	SO_0444 family Cu/Zn efflux transporter [Acidihalobacter prosperus]	93.85%	WP_052064744.1	SO_0444 family Cu/Zn efflux transporter [Acidithiobacillus thiooxidans]	70.13%	WP_051690760.1

2805	Transcriptional regulator, ArsR family CDS	winged helix-turn-helix transcriptional regulator [Acidihalobacter prosperus]	100%	WP_070079210.1	metalloregulator ArsR/SmtB family transcription factor [Acidithiobacillus thiooxidans]	70.13%	WP_155270561.1
2806	Hypothetical protein	cation transporter [Acidihalobacter prosperus]	89.19%	WP_052064742.1	cation transporter [Leptospirillum ferriphilum]	70.13%	WP_082317916.1
2807	Cobalt-zinc-cadmium resistance protein CzcD CDS	hypothetical protein [Acidihalobacter prosperus]	100%	WP_156782764.1	cation efflux system protein [Leptospirillum ferrooxidans]	70.13%	AEN14653.1
2808	Mobile element protein	IS5 family transposase [Acidihalobacter prosperus]	98.34	WP_070079198.1	IS5 family transposase [Pseudomonas aeruginosa]	88.40%	WP_121335850.1
2809	Mobile element protein	IS5 family transposase [Acidihalobacter prosperus]	100%	WP_070078194.1	IS5 family transposase [Xanthomonas cassavae]	82.76%	WP_029218204.1
2810	cytochrome c biogenesis protein, transmembrane region CDS	hypothetical protein BI364_13610 [Acidihalobacter prosperus]	100%	AOU98856.1	hypothetical protein EPN38_07435 [Rhodanobacteraceae bacterium]	63.04%	TAN06685.1
2811	Lead, cadmium, zinc and mercury transporting ATPase (EC 3.6.3.3) (EC 3.6.3.5); Copper-translocating P-type	cadmium-translocating P-type ATPase [Acidihalobacter prosperus] 100%		WP_070079212.1	copper-translocating P-type ATPase [Rhodanobacter sp. SCN 69-32]	57.47%	ODU74320.1

	ATPase (EC 3.6.3.4) CDS						
2812	Hypothetical protein	No significant similarity found	100%	WP_070079213.1	Unique	unique	unique
2813	Mobile element protein	No significant similarity found	0%		IS630 family transposase [Thiomonas sp. 14-64-326]	89.10%	OZB68769.1

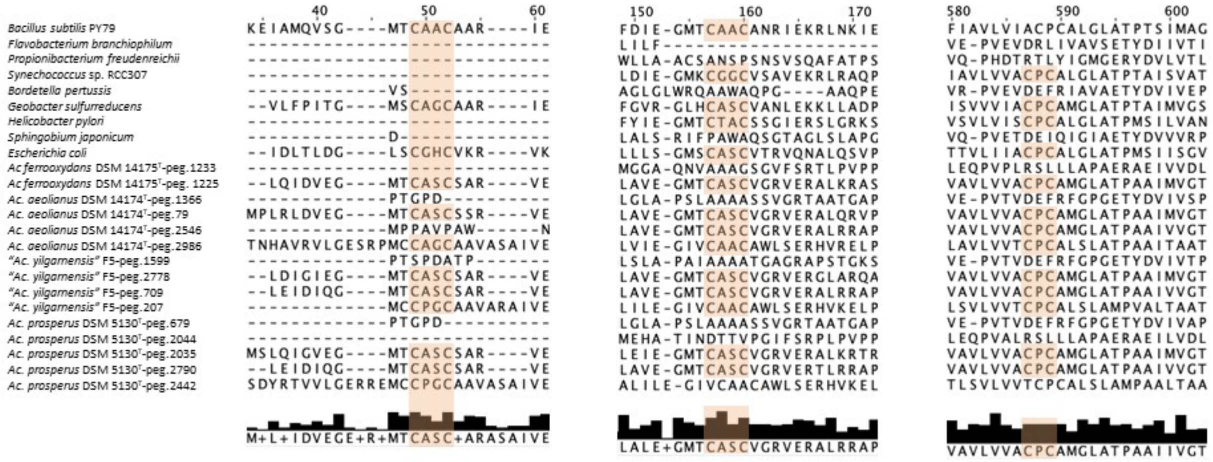

Figure S1. Identification of the metal binding motifs for potential *CopA* proteins in the *Acidihalobacter* genomes compared with copA sequences from members of Firmicutes (*Bacillus subtilis*), CFB group bacteria (*Flavobacterium branchiophilum*), Actinobacteria (*Propionibacterium freudenreichii*), Cyanobacteria (*Synechococcus sp RCC307*), Betaproteobacteria (*Bordetella pertussis*), Deltaproteobacteria (*Geobacter sulfurreducens*), Epsilonproteobacteria (*Helicobacter pylori*), Alphaproteobacteria (*Sphingobium japonicum*) and Gammaproteobacteria (*Escherichia coli*).

Table S2. BLASTx analysis of the sequences of the catalase genes for *Ac. yilgarnensis* F5^T, *Ac. prosperus* DSM 5130^T and *Ac. ferrooxydans* DSM 14175^T. Homologs of the catalase genes were not found in the genome of *Ac. aeolianus* DSM 14174$^{T.}$

**Genome**	**RAST annotation**	**NCBI annotation**	**Ident (%) to *Acidhalobacter* sp.**	**NCBI acc.**	**Next best hit (NBH)**	**Protein Ident (%) to NBH**	**NCBI acc.**
Acidihalobacter yilgarnensis F5^T	Catalase (EC 1.11.1.6) / Peroxidase (EC 1.11.1.7) CDS	Catalase/ peroxidase HPI [Acidihalobacter prosperus]	100%	WP_070079485.1	catalase/peroxidase HPI [Sulfuricaulis limicola]	77.39%	WP_096359101.1
Acidihalobacter prosperus DSM 5130^T	Catalase (EC 1.11.1.6) / Peroxidase (EC 1.11.1.7) CDS	catalase/ peroxidase HPI [Acidihalobacter prosperus]	100%	WP_038090519.1	catalase/peroxidase HPI [Sulfuricaulis limicola]	77.90%	WP_096359101.1
Acidihalobacter ferrooxydans DSM 14175^T	Catalase (EC 1.11.1.6) / Peroxidase (EC 1.11.1.7) CDS	catalase/ peroxidase HPI [uncultured bacterium UPO76]	79.09%	AMK59609.1	catalase/peroxidase HPI [Nitratireductor sp. StC3]	76.70%	WP_106571389.1

Supplementary File 2.

Unlocking survival mechanisms for metal and oxidative stress in the extremely acidophilic, halotolerant *Acidihalobacter* genus

Himel Nahreen Khaleque[1,5], Homayoun Fathollazadeh[1], Carolina González[2,4], Raihan Shafique[1], Anna H. Kaksonen[5], David S. Holmes[2,3,4] and Elizabeth L.J. Watkin[1*]

[1]School of Pharmacy and Biomedical Sciences, Curtin University, Perth, Australia; [2]Center for Bioinformatics and Genome Biology, Fundacion Ciencia y Vida, Santiago, Chile; [3]Universidad San Sebastian, Santiago Chile; [4]Centro de Genómica y Bioinformática, Facultad de Ciencias, Universidad Mayor, Santiago, Chile; and [5]CSIRO Land and Water, Floreat, Australia

*Correspondence: E.Watkin@curtin.edu.au; Tel.: (+61 8 92662955)

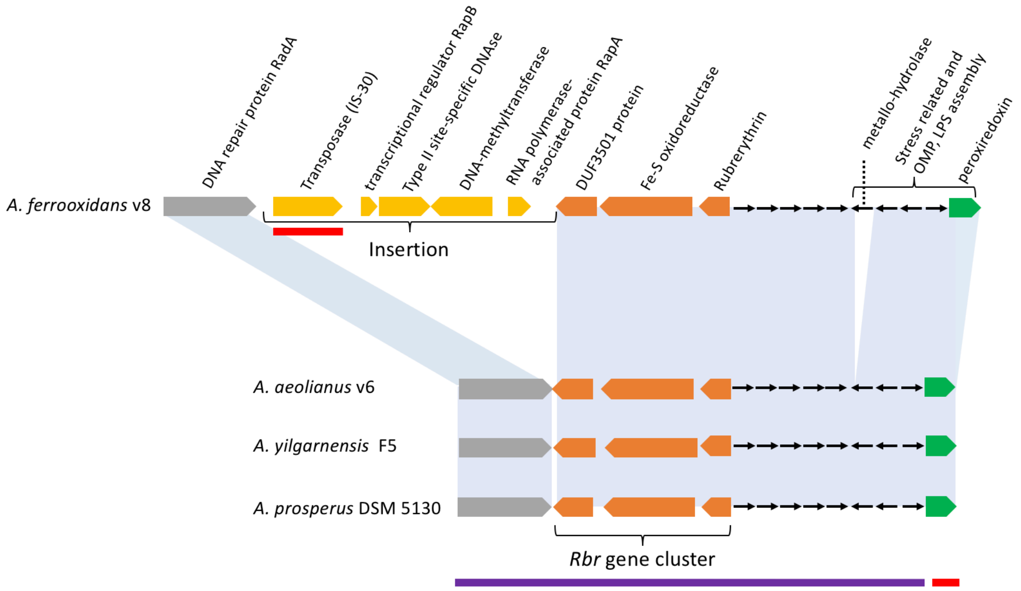

Taken from Figure 3 of the above paper. Genomic context of rubrerythrin (*rbr*) and neighboring genes in the four species of *Acidihalobacter*. Red underlining = genes with similarity to orthologs in acidophiles; purple underlining = genes with similarity to orthologs in neutrophilic, alkalophilic, halophilic and halotolerant organisms and other non-acidophiles

All BlastP analyses were carried out using default parameters. Only sequences corresponding to proteins predicted in *Acidihalobacter ferrooxydans* are shown in the additional file.

In the following pages, we provide additional information about some of the genes shown in the Figure above.

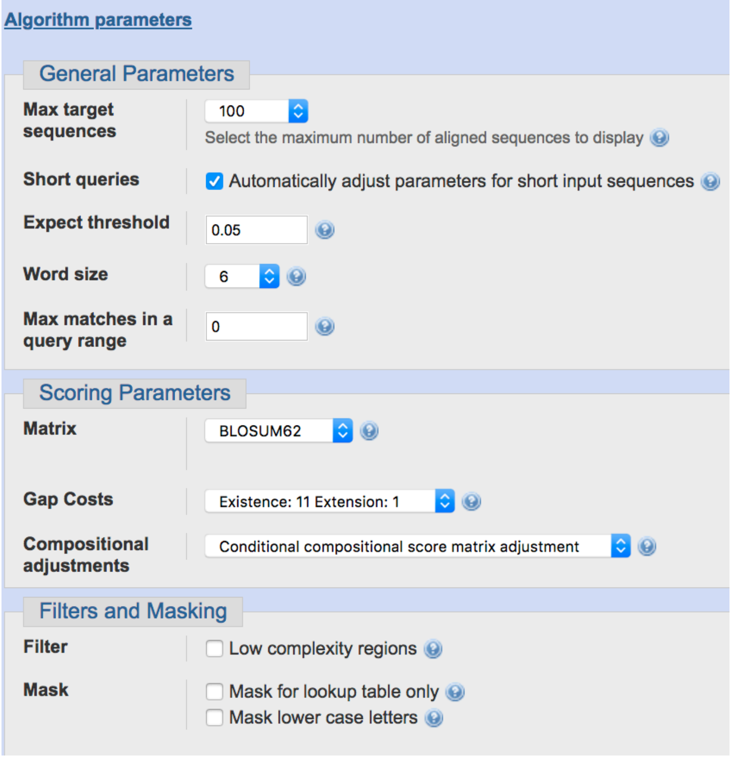

Altschul, S.F., Gish, W., Miller, W., Myers, E.W. & Lipman, D.J. (1990) "Basic local alignment search tool." J. Mol. Biol. 215:403-410.
Marchler-Bauer A et al. (2017), "CDD/SPARCLE: functional classification of proteins via subfamily domain architectures.", Nucleic Acids Res. 45(D)200-3.

DNA repair protein RadA

>WP_076837516.1 DNA repair protein RadA [*Acidihalobacter ferrooxidans*]
MAKARVEYRCNACGAFSPKWSGQCGECGEWNTLVEVAPRKTSAPPPPNPRFSGYAGHSSVRKMDEV
ALEAEPRTPTRLTEFDRVLGGGLVHGSVVLIGGDPGIGKSTLLLQTLATLDKSLPSLYVTGEESLQQVTLRA
HRLGLPRGHLRLLTETGVERILAIATEERPRVLVIDSIQTLYSEQLQSAPGSIGQVRESAAQLVRYAKQTDTS
VFLVGHVTKEGTLAGPRVLEHMVDTVLYFEGDPGNRYRVMRAVKNRFGAVNELGVFAMTEHGLKAVS
NPYTQAIVPKANAPRHTSAGMQVHAVERLSEALEKLL

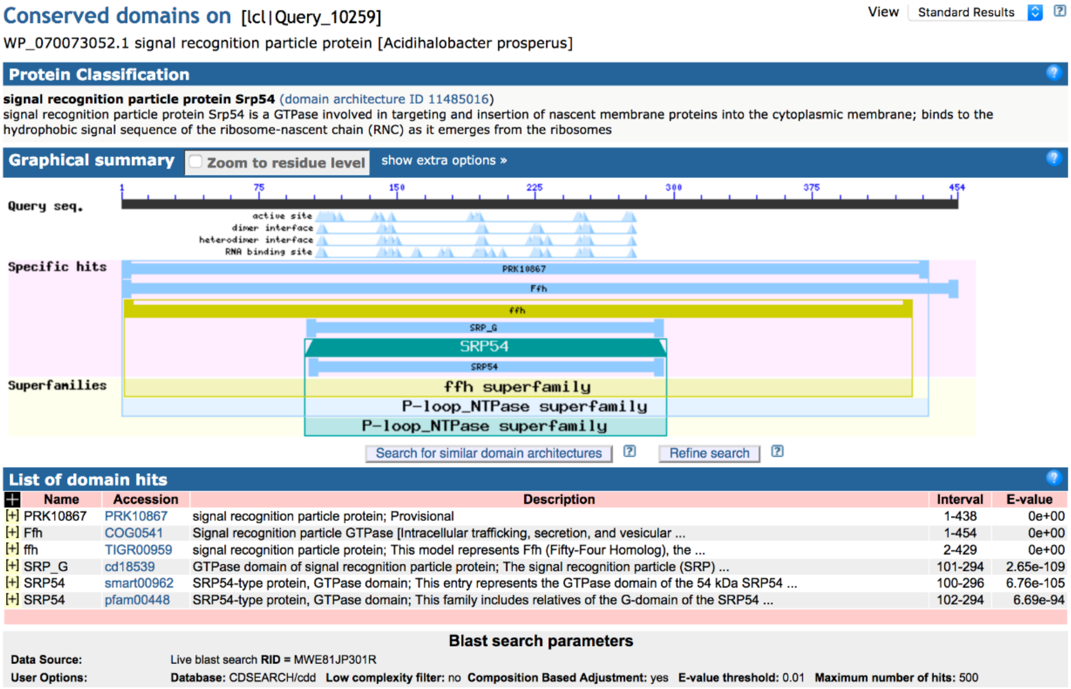

Conserved domains on [lcl|Query_10259]
WP_070073052.1 signal recognition particle protein [Acidihalobacter prosperus]

Protein Classification

signal recognition particle protein Srp54 (domain architecture ID 11485016)
signal recognition particle protein Srp54 is a GTPase involved in targeting and insertion of nascent membrane proteins into the cytoplasmic membrane; binds to the hydrophobic signal sequence of the ribosome-nascent chain (RNC) as it emerges from the ribosomes

List of domain hits

Name	Accession	Description	Interval	E-value
[+] PRK10867	PRK10867	signal recognition particle protein; Provisional	1-438	0e+00
[+] Ffh	COG0541	Signal recognition particle GTPase [Intracellular trafficking, secretion, and vesicular ...	1-454	0e+00
[+] ffh	TIGR00959	signal recognition particle protein; This model represents Ffh (Fifty-Four Homolog), the ...	2-429	0e+00
[+] SRP_G	cd18539	GTPase domain of signal recognition particle protein; The signal recognition particle (SRP) ...	101-294	2.65e-109
[+] SRP54	smart00962	SRP54-type protein, GTPase domain; This entry represents the GTPase domain of the 54 kDa SRP54 ...	100-296	6.76e-105
[+] SRP54	pfam00448	SRP54-type protein, GTPase domain; This family includes relatives of the G-domain of the SRP54 ...	102-294	6.69e-94

Blast search parameters

Data Source: Live blast search RID = MWE81JP301R
User Options: **Database:** CDSEARCH/cdd **Low complexity filter:** no **Composition Based Adjustment:** yes **E-value threshold:** 0.01 **Maximum number of hits:** 500

Next best BlastP hits after *Acidihalobacter*:

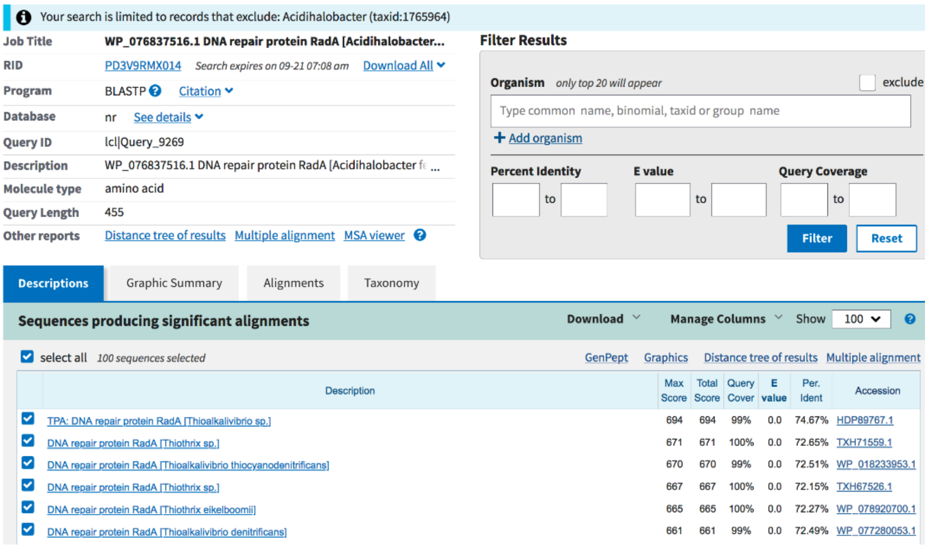

Your search is limited to records that exclude: Acidihalobacter (taxid:1765964)

Job Title	WP_076837516.1 DNA repair protein RadA [Acidihalobacter...
RID	PD3V9RMX014 Search expires on 09-21 07:08 am Download All
Program	BLASTP Citation
Database	nr See details
Query ID	lcl|Query_9269
Description	WP_076837516.1 DNA repair protein RadA [Acidihalobacter f...
Molecule type	amino acid
Query Length	455
Other reports	Distance tree of results Multiple alignment MSA viewer

Sequences producing significant alignments

Description	Max Score	Total Score	Query Cover	E value	Per. Ident	Accession
TPA: DNA repair protein RadA [Thioalkalivibrio sp.]	694	694	99%	0.0	74.67%	HDP89767.1
DNA repair protein RadA [Thiothrix sp.]	671	671	100%	0.0	72.65%	TXH71559.1
DNA repair protein RadA [Thioalkalivibrio thiocyanodenitrificans]	670	670	99%	0.0	72.51%	WP_018233953.1
DNA repair protein RadA [Thiothrix sp.]	667	667	100%	0.0	72.15%	TXH67526.1
DNA repair protein RadA [Thiothrix eikelboomii]	665	665	100%	0.0	72.27%	WP_078920700.1
DNA repair protein RadA [Thioalkalivibrio denitrificans]	661	661	99%	0.0	72.49%	WP_077280053.1

Thioalkalivibrio is a Gram negative bacterial genus of the family Ectothiorhodospiraceae. They are chemolithoautotrophic, haloalkaliphilic sulfur-oxidizing bacteria (Seckbach, Joseph, Aharon Oren, and Helga Stan-Lotter. "Polyextremophiles." *Dordrecht: Springer. doi* 10 (2013): 978-94).

IS30 family transposase

>WP_076837362.1 IS30 family transposase [*Acidihalobacter ferrooxidans*]
MGSRYKQLSMDERNRLQRGLNQGMSLRALARALGRHVSTLSRECRRGWIGSSYDAVQGREAARIRRR
RGTRKLLAGSPLADLVAWQIIQHAWSPEQIAGRLRMEHPEEARQRVSHETIYQFIYAHPAGALKKLLVES
LRQGHQKRRPRRRGQDRRGSLRNMRSIRERPEEAQAREIPGHWEADLIKGAYNGSAIGTLVDRSTRFTL
LARVDDSSAEAVLDGFTRRLRTLPKALRKTLTYDQGKEMARHEELEKRTHLRVYFADPHSPWQRPTNEN
THGLLRQYFPKGTDLSLYSQQYLTKVAEELNNRPRKTLGFRTPAEVMAEKISALNRSVALQN

Conserved domains on [lcl|Query_26943] View Standard Results

WP_076837362.1 IS30 family transposase [Acidihalobacter ferrooxidans]

Protein Classification

Warning: This architecture is curated on proteins in taxonomy scope **eubacteria**
IS30 family transposase (domain architecture ID 11458135)
IS30 family transposase binds to the end of a transposon and catalyzes the movement of the transposon to another part of the genome by a cut and paste mechanism or a replicative transposition mechanism

Graphical summary Zoom to residue level show extra options »

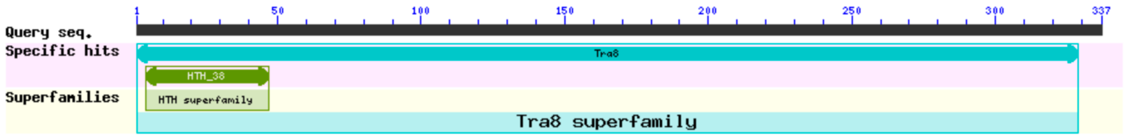

Search for similar domain architectures Refine search

List of domain hits

Name	Accession	Description	Interval	E-value
[+] Tra8	COG2826	Transposase and inactivated derivatives, IS30 family [Mobilome: prophages, transposons];	1-329	1.72e-98
[+] HTH_38	pfam13936	Helix-turn-helix domain; This helix-turn-helix domain is often found in transferases and is ...	4-47	6.51e-15

Next best BlastP hits after *Acidihalobacter*:

Description	Max Score	Total Score	Query Cover	E value	Per. Ident	Accession
IS30 family transposase [Acidithiobacillus ferrooxidans]	539	539	100%	0.0	77.58%	WP_163053951.1
IS30 family transposase [Acidithiobacillus thiooxidans]	503	503	100%	4e-177	71.51%	WP_153940591.1
IS30-like element IST3091 family transposase [Acidithiobacillus ferrooxidans]	501	501	99%	3e-176	69.64%	WP_054608914.1
IS30 family transposase [Acidithiobacillus sp. SH]	499	499	100%	3e-175	70.92%	WP_101536841.1
IS30-like element ISCARN114 family transposase [Acidithiobacillus thiooxidans]	488	977	100%	4e-171	70.92%	WP_065974143.1
MULTISPECIES: IS30-like element ISCARN114 family transposase [Acidithiobacillus]	484	484	100%	1e-169	70.62%	WP_040429107.1
IS30-like element IST3091 family transposase [Acidithiobacillus ferrooxidans]	483	483	95%	5e-169	70.40%	WP_074874147.1
IS30-like element ISCARN114 family transposase [Acidithiobacillus albertensis]	482	482	100%	1e-168	70.33%	WP_075324157.1
IS30-like element ISCARN114 family transposase [Acidithiobacillus thiooxidans]	481	481	100%	2e-168	70.33%	WP_153940740.1
IS30 family transposase [Acidithiobacillus ferridurans]	455	455	99%	6e-158	66.96%	WP_011117673.1
IS30-like element ISCARN114 family transposase [Acidithiobacillus thiooxidans]	418	418	77%	2e-144	74.81%	WP_081334431.1
hypothetical protein A4U49_06315 [Acidithiobacillus ferrivorans]	412	825	74%	2e-142	77.29%	OFA16659.1
hypothetical protein A2514_09870 [Gammaproteobacteria bacterium RIFOXYD12_FULL_61_37]	401	401	100%	1e-136	61.54%	OGT89220.1
hypothetical protein A4U49_15525 [Acidithiobacillus ferrivorans]	391	391	64%	2e-133	83.56%	OFA14986.1
IS30 family transposase [Acidithiobacillus ferrooxidans]	390	390	79%	4e-133	68.15%	WP_163059866.1
hypothetical protein A6O26_01650 [Acidithiobacillus thiooxidans]	384	769	69%	1e-131	76.17%	OCX85176.1

Some background information about IS30 family transposases.

Lysnyansky, Inna, et al. "Molecular characterization of newly identified IS 3, IS 4 and IS 30 insertion sequence-like elements in Mycoplasma bovis and their possible roles in genome plasticity." *FEMS microbiology letters* 294.2 (2009): 172-182.
Dong, Q., et al. "Cloning and sequencing of IS1086, an Alcaligenes eutrophus insertion element related to IS30 and IS4351." *Journal of bacteriology* 174.24 (1992): 8133-8138.

IS30 involvement in metal resistance:

Janssen, Paul J., et al. "The complete genome sequence of Cupriavidus metallidurans strain CH34, a master survivalist in harsh and anthropogenic environments." *PLoS One* 5.5 (2010): e10433.
Mergeay, Max, et al. "Ralstonia metallidurans, a bacterium specifically adapted to toxic metals: towards a catalogue of metal-responsive genes." *FEMS microbiology reviews* 27.2-3 (2003): 385-410.

IS30 involvement in oxidative stress:

Wright, Meredith S., et al. "Assessment of insertion sequence mobilization as an adaptive response to oxidative stress in Acinetobacter baumannii using IS-seq." *Journal of bacteriology* 199.9 (2017).

Helix-turn-helix domain-containing protein HipB-like

>BW247_12995 [*Acidihalobacter ferrooxidans*]
MVRKSSDSLRAILAENIKTFRREKGFSQEELAERCGLHRTYIGSVERHERNVTLSTLEVL
ASTLGVIVPELLTE

Note: this gene is incorrectly annotated in the NCBI database.

Conserved domains on [lcl|Query_1174]

View Standard Results

BW247_12995

Protein Classification

helix-turn-helix transcriptional regulator (domain architecture ID 10054599)
helix-turn-helix (HTH) transcriptional regulator containing a XRE family HTH domain, such as the major virulence regulator Bacillus PlcR and Streptococcus thermophilus ComR, which positively controls competence for natural DNA transformation

Graphical summary Zoom to residue level show extra options »

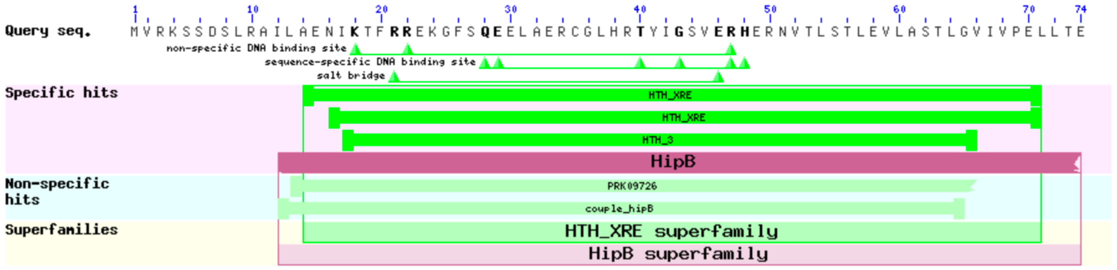

Search for similar domain architectures Refine search

List of domain hits

Name	Accession	Description	Interval	E-value
[+] HTH_XRE	cd00093	Helix-turn-helix XRE-family like proteins. Prokaryotic DNA binding proteins belonging to the ...	14-71	3.50e-12
[+] HTH_XRE	smart00530	Helix-turn-helix XRE-family like proteins;	16-71	2.13e-11
[+] HTH_3	pfam01381	Helix-turn-helix; This large family of DNA binding helix-turn helix proteins includes Cro and ...	17-66	4.01e-10
[+] PRK09726	PRK09726	type II toxin-antitoxin system antitoxin HipB;	13-66	2.46e-06
[+] HipB	COG1396	Transcriptional regulator, contains XRE-family HTH domain [Transcription];	12-74	6.51e-05
[+] couple_hipB	TIGR03070	transcriptional regulator, y4mF family; Members of this family belong to a clade of ...	12-65	2.67e-03

Blast search parameters

Next best BlastP hits after *Acidihalobacter*:

Alignments Download GenPept Graphics Distance tree of results Multiple alignment

Description	Max Score	Total Score	Query Cover	E value	Per. Ident	Accession
helix-turn-helix transcriptional regulator [Candidatus Accumulibacter phosphatis]	143	143	100%	6e-43	95.95%	WP_034947003.1
helix-turn-helix transcriptional regulator [Methylobacillus flagellatus]	141	141	100%	4e-42	91.89%	WP_137718730.1
helix-turn-helix domain-containing protein [Acidihalobacter ferrooxidans]	149	149	100%	6e-42	100.00%	WP_083700248.1
helix-turn-helix transcriptional regulator [Caballeronia glebae]	136	136	100%	4e-40	91.89%	WP_086973940.1
XRE family transcriptional regulator [Leptolyngbya sp.]	134	134	100%	2e-39	89.19%	PZV06891.1
helix-turn-helix transcriptional regulator [Burkholderia novacaledonica]	134	134	100%	2e-39	89.19%	WP_106855686.1

Site specific Nuclease SmaI superfamily

>APZ44708.1 hypothetical protein BW247_13000 [*Acidihalobacter ferrooxidans*]
MGKPVVSISEKEISEIVEPLRRLTPAQQAWVKSAIRAFGTACQFNRAPDSDVVTDAVLASLGDRLLSHHA
GSRQALSKDRFEFAFEAALNASGITAKLVKSRTNRGHDITIRGIPVSLKTEAAANIKDESIHVSKWMELGR
GEWKLPLLRDLFLEHMQSYDRIFTLRRLKDAGAKTRYELVEIPKELLLEAANCELEVCSSSKQNPQPGYGY
VKDADGQLKYSLYFDGGTERKLQIKHLRKDLCKVHATWIFGSTPA

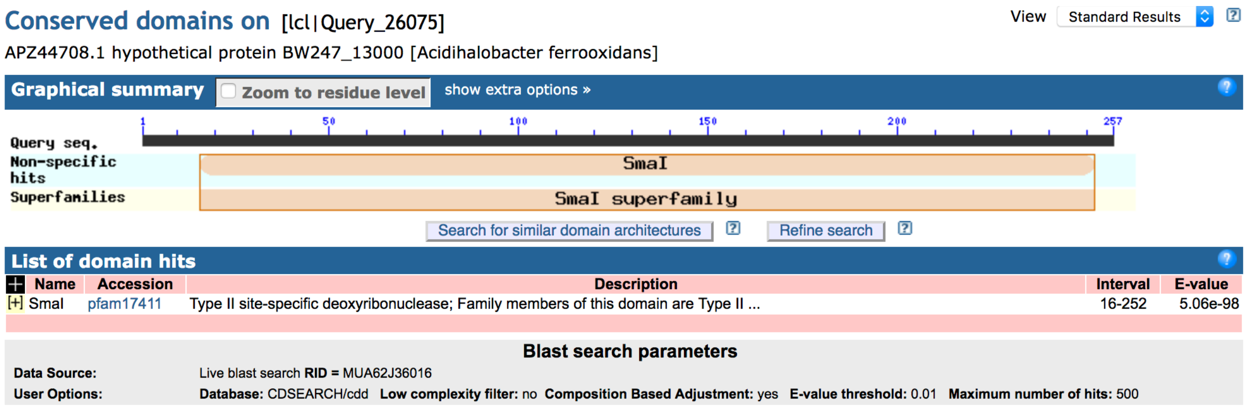

Withers, Barbara E., and Joan C. Dunbar. "Sequence-specific DNA recognition by the SmaI endonuclease." *Journal of Biological Chemistry* 270.12 (1995): 6496-6504.

Next best BlastP hits after *Acidihalobacter*:

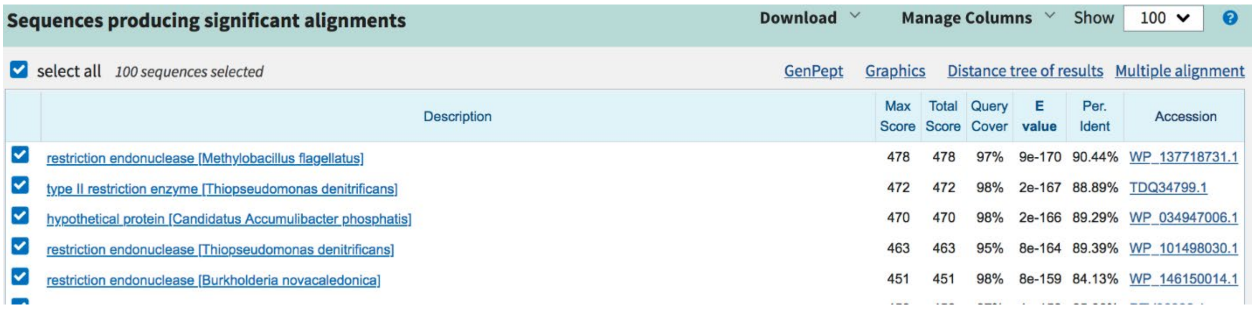
Sequences producing significant alignments — Download — Manage Columns — Show 100

select all 100 sequences selected — GenPept — Graphics — Distance tree of results — Multiple alignment

Description	Max Score	Total Score	Query Cover	E value	Per. Ident	Accession
restriction endonuclease [Methylobacillus flagellatus]	478	478	97%	9e-170	90.44%	WP_137718731.1
type II restriction enzyme [Thiopseudomonas denitrificans]	472	472	98%	2e-167	88.89%	TDQ34799.1
hypothetical protein [Candidatus Accumulibacter phosphatis]	470	470	98%	2e-166	89.29%	WP_034947006.1
restriction endonuclease [Thiopseudomonas denitrificans]	463	463	95%	8e-164	89.39%	WP_101498030.1
restriction endonuclease [Burkholderia novacaledonica]	451	451	98%	8e-159	84.13%	WP_146150014.1

Methylobacillus is a group of methylotrophic aerobic bacteria, and they can be found in large numbers in marine and fresh water ecosystems. Chistoserdova, Ludmila, et al. "Genome of Methylobacillus flagellatus, molecular basis for obligate methylotrophy, and polyphyletic origin of methylotrophy." *Journal of bacteriology* 189.11 (2007): 4020-4027.

Tan, Wen-Bo, et al. "*Thiopseudomonas* denitrificans gen. nov., sp. nov., isolated from anaerobic activated sludge." *International journal of systematic and evolutionary microbiology* 65.1 (2015): 225-229. oxidize sulfide anaerobically with nitrate as electron acceptor. The
strain grew at salinities of 0–3%(w/v) NaCl (optimum, 0–1%). Growth occurred at pH 6.0–10.0 (optimum, pH 8.0) and 10–37° C (optimum, 30° C). The genomic DNA G+ C content was 59 mol%.

Predicted site-specific DNA methyltransferase

>WP_076837517.1 site-specific DNA-methyltransferase [*Acidihalobacter ferrooxidans*]
MNETTQILDLFDTGGKALGAQLAEGTANRCELLVGDARQLLSRMPDGHFDCIVTSPPYWGLRDYGVQG
QI
GAEPTVDDYIADLVRLFREVRRTLSDEGTLWLNIGDSYTSGGRTWRDADAKNKGRAMDYRAPTPEGLK
PK
DLIGVPWRLAFALQADGWYLRTDIIWNKPNCQPESVKDRPTRAHEYVFLFSKSEKYYYDWQAIMEPATN
P
RQKSKNRRTVWNINTEPYPGSHFAVYPKALVRMCVEAGSSKNGRVLDPFFGSGTTGVVCNELGRDCVG
IE
LSAEYAELARERLLRGR

Protein Classification

site-specific DNA-methyltransferase (domain architecture ID 10480401)
site-specific DNA-methyltransferase catalyzes specific methylation on cytosine or adenine and protects the DNA from cleavage by endonuclease

Graphical summary Zoom to residue level show extra options »

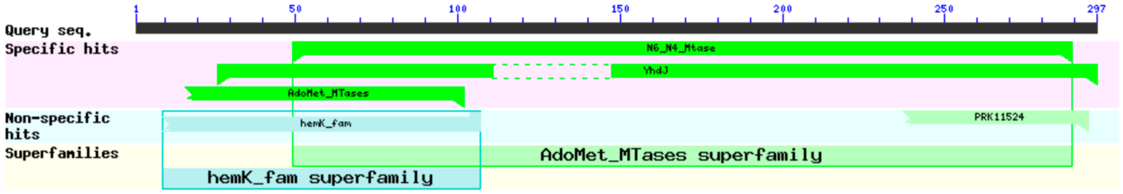

Search for similar domain architectures | Refine search

List of domain hits

Name	Accession	Description	Interval	E-value
[+] N6_N4_Mtase	pfam01555	DNA methylase; Members of this family are DNA methylases. The family contains both N-4 ...	49-289	2.02e-59
[+] YhdJ	COG0863	DNA modification methylase [Replication, recombination and repair];	26-297	6.27e-32
[+] PRK11524	PRK11524	adenine-specific DNA-methyltransferase;	237-294	4.34e-09
[+] hemK_fam	TIGR00536	HemK family putative methylases; The gene hemK from E. coli was found to contribute to heme ...	9-107	1.18e-03
[+] AdoMet_MTases	cd02440	S-adenosylmethionine-dependent methyltransferases (SAM or AdoMet-MTase), class I; ...	16-102	2.94e-03

Next best BlastP hits after *Acidihalobacter*:

Description	Max Score	Total Score	Query Cover	E value	Per. Ident	Accession
site-specific DNA-methyltransferase [Methylobacillus flagellatus]	576	576	100%	0.0	92.59%	WP_137718732.1
site-specific DNA-methyltransferase [Thiopseudomonas denitrificans]	556	556	100%	0.0	87.21%	WP_101498029.1
site-specific DNA-methyltransferase [Leptolyngbya sp.]	555	555	100%	0.0	88.22%	PZV06893.1
site-specific DNA-methyltransferase [Candidatus Accumulibacter phosphatis]	554	554	100%	0.0	87.21%	WP_138678593.1
site-specific DNA-methyltransferase [Caballeronia glebae]	546	546	100%	0.0	85.86%	WP_086973932.1
gp56 [Caballeronia glebae]	545	545	100%	0.0	85.86%	SAK96717.1
site-specific DNA-methyltransferase [Alcaligenaceae bacterium]	539	539	100%	0.0	84.51%	WP_179987460.1
MULTISPECIES: site-specific DNA-methyltransferase [Gammaproteobacteria]	538	538	98%	0.0	86.64%	WP_106442997.1
modification methylase [Gammaproteobacteria bacterium RIFOXYA12_FULL_61_12]	537	537	95%	0.0	89.44%	OGT88722.1
site-specific DNA-methyltransferase [Burkholderia novacaledonica]	535	535	100%	0.0	84.18%	WP_106855688.1
site-specific DNA-methyltransferase [Melaminivora sp. SC2-9]	533	533	100%	0.0	85.19%	WP_106684007.1

Hypothetical protein with truncated RapA-like domain

>WP_076837518.1 hypothetical protein [Acidihalobacter ferrooxidans]
MDVFGSKTQKLLADAQGLLQQAQHEMSKDIEKLKAKQSATDARLARLENQQDNSAREIGRLQQSLDDI
RD
SAIRGEIASGQKTKDVADKFNLTPARVSQIAPRRKYNNG

Conserved domains on [lcl|Query_27062]

View Standard Results

BW247_13010 RNA hypothetical protein (truncated polymerase-associated protein RapA)

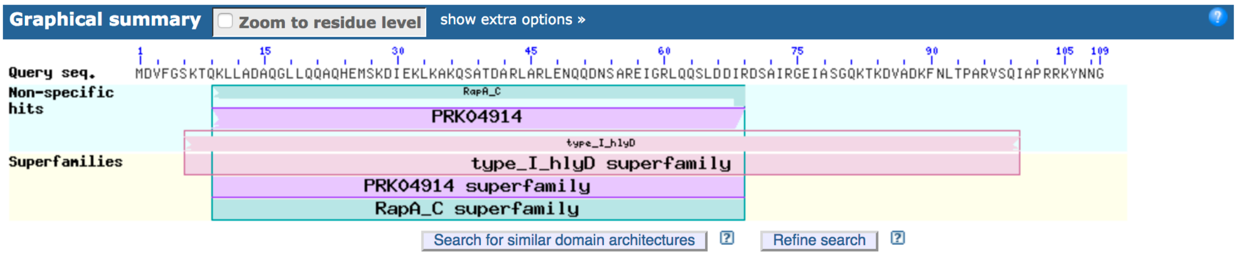

List of domain hits

Name	Accession	Description	Interval	E-value
[+] RapA_C	pfam12137	RNA polymerase recycling family C-terminal; This domain is found in bacteria. This domain is ...	9-69	7.18e-03
[+] PRK04914	PRK04914	RNA polymerase-associated protein RapA;	9-69	7.36e-03
[+] type_I_hlyD	TIGR01843	type I secretion membrane fusion protein, HlyD family; Type I secretion is an ABC transport ...	6-100	8.35e-03

Next best BlastP hits after *Acidihalobacter*:

Sequences producing significant alignments:

Select: All None Selected:0

Alignments Download GenPept Graphics Distance tree of results Multiple alignment

Description	Max Score	Total Score	Query Cover	E value	Per. Ident	Accession
hypothetical protein [Gammaproteobacteria bacterium]	181	181	100%	5e-57	89.91%	NLD15601.1
hypothetical protein [Aeromonas veronii]	166	166	100%	4e-51	73.39%	WP_139742379.1
hypothetical protein A6763_19535 [Aeromonas caviae]	166	166	100%	8e-51	72.48%	OCW44990.1
hypothetical protein [Enterobacter asburiae]	165	165	100%	2e-50	73.39%	WP_105629798.1
MULTISPECIES: hypothetical protein [Klebsiella]	158	158	100%	6e-48	70.64%	WP_071739097.1
hypothetical protein [Paraburkholderia phenazinium]	149	149	100%	4e-44	65.49%	WP_122153148.1

Transcription regulator that activates transcription by stimulating RNA polymerase (RNAP) recycling in case of stress conditions such as supercoiled DNA or high salt concentrations. Probably acts by releasing the RNAP, when it is trapped or immobilized on tightly supercoiled DNA. Does not activate transcription on linear DNA. Probably not involved in DNA repair (By similarity).

Sukhodolets, Maxim V., et al. "RapA, a bacterial homolog of SWI2/SNF2, stimulates RNA polymerase recycling in transcription." *Genes & development* 15.24 (2001): 3330-3341.

N-terminal Truncated RapA: Kakar, Smita, et al. "Allosteric activation of bacterial Swi2/Snf2 (Switch/Sucrose non-fermentable) protein RapA by RNA polymerase biochemical and structural studies." *Journal of Biological Chemistry* 290.39 (2015): 23656-23669.
Members of the Swi2/Snf2 (switch/sucrose non-fermentable) family depend on their ATPase activity to mobilize nucleic acid-protein complexes for gene expression. We conclude that the ATPase activity of RapA is inhibited by its Ntd but stimulated by RNAP in an allosteric fashion and that the conformational changes of RapA and its interaction with RNAP are essential for RNAP recycling.

DUF3501 family protein

>WP_076837519.1:3-193 DUF3501 family protein [Acidihalobacter ferrooxidans]
KLTRADLMSLETYATRRPEFRAQVMAHKRARRLALGPHAVLYFEDRLTIQYQVQEMLRAERIFEAAGIVD
ELDAYNPLIPDGSNWKATFMLEYTEAAERHEALKRLIGIEDRVWMRVEGFDKVYAIADEDLERETEEKTS
TVHFVRFELSAGMVAAAKGGAPISGGIDHKAMPLVVDPLPEATALSLVADL

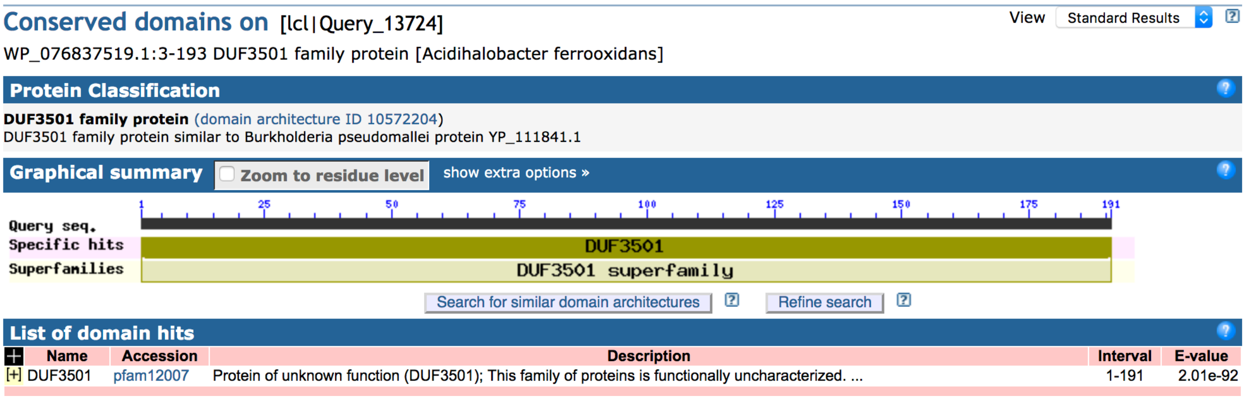

Name	Accession	Description	Interval	E-value
DUF3501	pfam12007	Protein of unknown function (DUF3501); This family of proteins is functionally uncharacterized. ...	1-191	2.01e-92

Next best BlastP hits after *Acidihalobacter*:

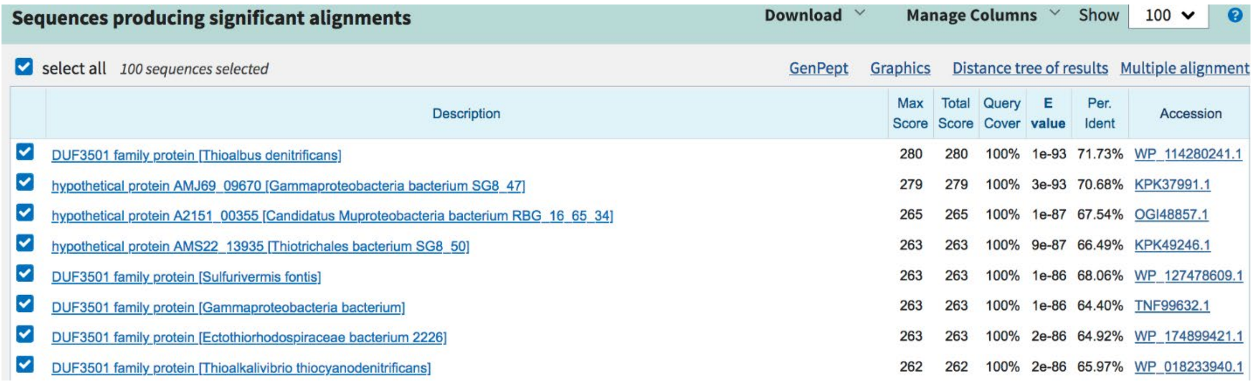

Description	Max Score	Total Score	Query Cover	E value	Per. Ident	Accession
DUF3501 family protein [Thioalbus denitrificans]	280	280	100%	1e-93	71.73%	WP_114280241.1
hypothetical protein AMJ69_09670 [Gammaproteobacteria bacterium SG8_47]	279	279	100%	3e-93	70.68%	KPK37991.1
hypothetical protein A2151_00355 [Candidatus Muproteobacteria bacterium RBG_16_65_34]	265	265	100%	1e-87	67.54%	OGI48857.1
hypothetical protein AMS22_13935 [Thiotrichales bacterium SG8_50]	263	263	100%	9e-87	66.49%	KPK49246.1
DUF3501 family protein [Sulfurivermis fontis]	263	263	100%	1e-86	68.06%	WP_127478609.1
DUF3501 family protein [Gammaproteobacteria bacterium]	263	263	100%	1e-86	64.40%	TNF99632.1
DUF3501 family protein [Ectothiorhodospiraceae bacterium 2226]	263	263	100%	2e-86	64.92%	WP_174899421.1
DUF3501 family protein [Thioalkalivibrio thiocyanodenitrificans]	262	262	100%	2e-86	65.97%	WP_018233940.1

Thioalbus is a mesophilic, facultatively anaerobic and autotrophic genus of bacteria from the family of Ectothiorhodospiraceae with one known species (*Thioalbus denitrificans*). *Thioalbus denitrificans* has been isolated from sediments from the Sea of Japan in Korea (Park, Soo-Je, et al. "Thioalbus denitrificans gen. nov., sp. nov., a chemolithoautotrophic sulfur-oxidizing gammaproteobacterium, isolated from marine sediment." *International journal of systematic and evolutionary microbiology* 61.9 (2011): 2045-2051).

Fe-S oxidoreductase

>WP_076837520.1:30-448 Fe-S oxidoreductase [*Acidihalobacter ferrooxidans*]
DHEALFAELERVYEICHGCRRCVSLCNAFPTLFDLVDESSTLEVDGVAKDDYWKVIDQCYLCDLCYMTKC
PYTPPHEWNVDFPHLMLRAKAVKYREGRTSKRDRLISSTDAIGRLAGIPVVAEAMNAANRNPAFREQLD
K
TMGIHAGALLPQYHSETGRQRVADHHSAVTQAVSAGRTQGRVAVFATCYGNYNEPHLIEDLFKVFEHN
GI
EMTLAEKERCCGMPKLELGDLEAVARAKEANIPVLARMIDAGWDIVGPVPSCVLMFKQELPLMFPDDP
DV
AKVKAHIFDPFEYLMLRQKEGLLNTEFKHSLGKIAYHAACHLRVQNMGLKTRELLELIPDTTLDVIERCS
GHDGTYAVKSEFHETSMKICRPVVTRVQKAESDHYSSDCPMAGHQIENGLKDERPPEHPLTLLRLAYGL

Conserved domains on [lcl|Query_19994] View Standard Results

WP_076837520.1:30-448 Fe-S oxidoreductase [Acidihalobacter ferrooxidans]

Graphical summary Zoom to residue level show extra options »

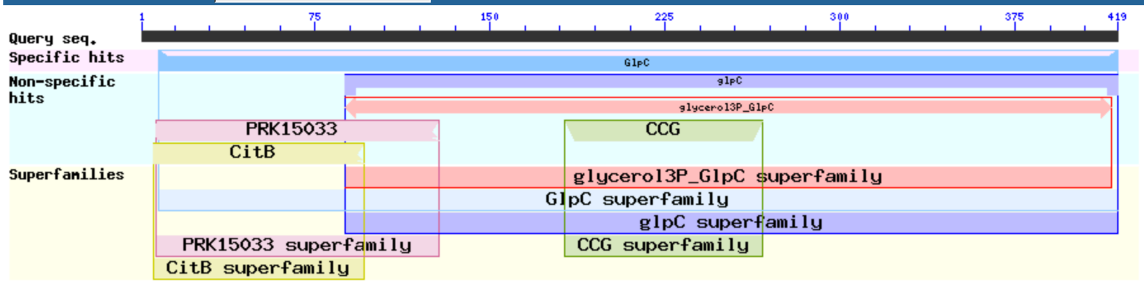

Search for similar domain architectures Refine search

List of domain hits

Name	Accession	Description	Interval	E-value
[+] glpC	PRK11168	anaerobic glycerol-3-phosphate dehydrogenase subunit C;	88-419	3.57e-78
[+] glycerol3P_GlpC	TIGR03379	glycerol-3-phosphate dehydrogenase, anaerobic, C subunit; Members of this protein family are ...	88-416	5.02e-42
[+] GlpC	COG0247	Fe-S oxidoreductase [Energy production and conversion];	8-419	7.69e-42
[+] PRK15033	PRK15033	tricarballylate utilization 4Fe-4S protein TcuB;	7-128	1.09e-07
[+] CitB	TIGR02484	CitB domain protein; This model identifies proteins of two distinct names which may or may not ...	6-96	4.85e-06
[+] CCG	pfam02754	Cysteine-rich domain; The key element of this family is the CX31-38CCX33-34CXXC sequence motif ...	182-267	3.76e-05

Next best BlastP hits after *Acidihalobacter*:

Sequences producing significant alignments Download Manage Columns Show 100

select all 100 sequences selected GenPept Graphics Distance tree of results Multiple alignment

Description	Max Score	Total Score	Query Cover	E value	Per. Ident	Accession
Fe-S oxidoreductase [Thioalkalivibrio thiocyanodenitrificans]	708	708	100%	0.0	78.76%	WP_018233938.1
Fe-S oxidoreductase [Thioalkalivibrio denitrificans]	696	696	100%	0.0	77.09%	WP_077279461.1
Fe-S oxidoreductase [Thioalkalivibrio sulfidiphilus]	689	689	100%	0.0	75.89%	WP_018953045.1
Fe-S oxidoreductase [Thioalkalivibrio sulfidiphilus]	687	687	100%	0.0	75.66%	WP_012637415.1
Fe-S oxidoreductase [Hydrogenophilales bacterium SM250]	677	677	100%	0.0	73.99%	WP_147799571.1
Fe-S oxidoreductase-like protein in Rubrerythrin cluster [uncultured Thiotrichaceae bacterium]	676	676	100%	0.0	72.55%	CAA6817167.1
Fe-S oxidoreductase [Thioalkalivibrio sp. ALJ16]	672	672	100%	0.0	73.93%	WP_018872777.1

Rubrerythrin

>WP_076837521.1:3-130 rubrerythrin [Acidihalobacter ferrooxidans]
LKGSKTEQSLKEAFSGESQANRRYLYFAAKADVEGQNDISAVFRSTAEGETGHAHGHLEYLEDCGDPATG
LPFGSTEANLKTAIAGETHEYTDMYPGMAKTAREEGFDEIADWFETLAKAERSHANRF

Conserved domains on [lcl|Query_30080] View Standard Results
WP_076837521.1:3-130 rubrerythrin [Acidihalobacter ferrooxidans]

Protein Classification

Rubrerythrin domain-containing protein (domain architecture ID 10099327)
Rubrerythrin domain-containing protein

Graphical summary Zoom to residue level show extra options »

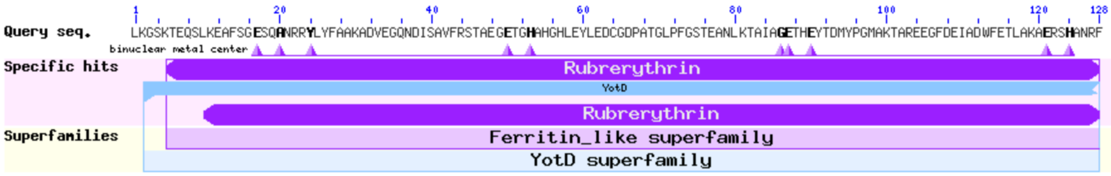

Search for similar domain architectures Refine search

List of domain hits

Name	Accession	Description	Interval	E-value
[+] Rubrerythrin	cd01041	Rubrerythrin, ferritin-like diiron-binding domain; Rubrerythrin domain is a nonheme iron ...	5-128	2.77e-56
[+] YotD	COG1592	Rubrerythrin [Energy production and conversion];	2-128	1.25e-39
[+] Rubrerythrin	pfam02915	Rubrerythrin; This domain has a ferritin-like fold.	10-128	1.53e-23

Next best BlastP hits after *Acidihalobacter*:

Sequences producing significant alignments:
Select: All None Selected:0
Alignments Download GenPept Graphics Distance tree of results Multiple alignment

Description	Max Score	Total Score	Query Cover	E value	Per. Ident	Accession
rubrerythrin [Thioalkalivibrio thiocyanodenitrificans]	253	253	100%	1e-84	94.53%	WP_018233937.1
rubrerythrin [Thioalkalivibrio sulfidiphilus]	253	253	100%	2e-84	93.75%	WP_018953044.1
rubrerythrin [Thioalkalivibrio sulfidiphilus]	253	253	100%	2e-84	93.75%	WP_012637414.1
rubrerythrin [Sulfurivermis fontis]	251	251	100%	4e-84	93.75%	WP_127478607.1
rubrerythrin [Thioalkalivibrio denitrificans]	251	251	100%	5e-84	93.75%	WP_077279460.1
TPA: rubrerythrin [Sedimenticola thiotaurini]	251	251	100%	1e-83	91.41%	HEB95199.1
TPA: rubrerythrin [Sedimenticola sp.]	250	250	100%	2e-83	91.41%	HHH38760.1
rubrerythrin [Thioalkalivibrio nitratireducens]	246	246	100%	4e-82	91.41%	WP_015260167.1

Berben, Tom, et al. "Comparative genome analysis of three thiocyanate oxidizing Thioalkalivibrio species isolated from soda lakes." *Frontiers in microbiology* 8 (2017): 254.

Sorokin, Dimitry Y., et al. "*Thioalkalivibrio sulfidiphilus* sp. nov., a haloalkaliphilic, sulfur-oxidizing gammaproteobacterium from alkaline habitats." *International journal of systematic and evolutionary microbiology* 62.8 (2012): 1884-1889.

Flood, Beverly E., Daniel S. Jones, and Jake V. Bailey. "*Sedimenticola thiotaurini* sp. nov., a sulfur-oxidizing bacterium isolated from salt marsh sediments, and emended descriptions of the genus Sedimenticola and Sedimenticola selenatireducens." *International journal of systematic and evolutionary microbiology* 65.8 (2015): 2522-2530.

MBL fold metallo-hydrolase

>APZ44710.1 MBL fold metallo-hydrolase
MRYASLGSGSRGNACVIESGGTRLLLDCGFSVREVDRRLARLGLRGEDIGAILITHEHSDHLSGAARLSRRY
RLPVWLTAGTLAACRDTGFYRIEQFHAHDVFAVGDIQLHPFPVPHDAREPAQFVFSDGDVRLGQLTDTG
SITPHIERMLAGVDALVLECNYEPELLRRGPYPPALKARVGGNYGHLGNDQAAALLARLDTSRLRWLIGA
HVSEKNNTPSHARAALIAGLGDDTERIAVAAQDEGSGWRSL

Conserved domains on [lcl|Query_16499] View Standard Results
APZ44710.1 MBL fold metallo-hydrolase

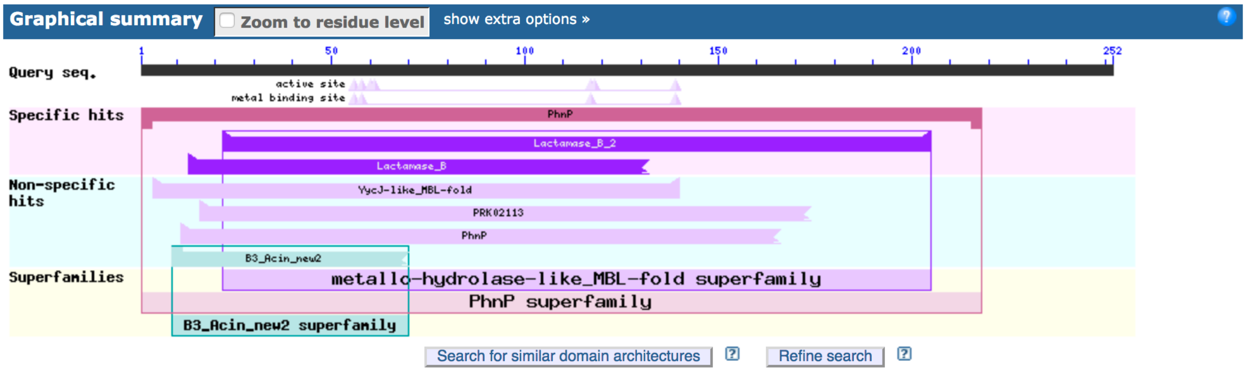

List of domain hits

Name	Accession	Description	Interval	E-value
[+] YycJ-like_MBL-fold	cd07733	uncharacterized subgroup which includes Bacillus subtilis YycJ and related proteins; MBL-fold ...	4-140	1.12e-46
[+] PhnP	COG1235	Phosphoribosyl 1,2-cyclic phosphodiesterase [Inorganic ion transport and metabolism];	1-218	8.20e-35
[+] Lactamase_B_2	pfam12706	Beta-lactamase superfamily domain; This family is part of the beta-lactamase superfamily and ...	22-205	1.38e-29
[+] Lactamase_B	smart00849	Metallo-beta-lactamase superfamily; Apart from the beta-lactamases a number of other proteins ...	13-132	9.30e-14
[+] PRK02113	PRK02113	MBL fold metallo-hydrolase;	16-174	1.46e-08
[+] PhnP	TIGR03307	phosphonate metabolism protein PhnP; This family of proteins found in operons encoding ...	11-166	1.48e-07
[+] B3_Acin_new2	NF033185	putative subclass B3 metallo-beta-lactamase; This is one of two families of putative ...	9-70	1.68e-03

Next best BlastP hits after *Acidihalobacter*:

MBL fold metallo-hydrolase [Thioalkalivibrio thiocyanodenitrificans]	317	317	100%	7e-106	61.51%	WP_018233931.1
MBL fold metallo-hydrolase [Thioalkalivibrio denitrificans]	311	311	99%	1e-103	62.40%	OOG23118.1
MBL fold metallo-hydrolase [Thioalkalivibrio denitrificans]	311	311	99%	2e-103	62.40%	WP_077279455.1
MBL fold metallo-hydrolase [Thioalkalivibrio sulfidiphilus]	307	307	99%	4e-102	60.40%	WP_018953038.1
MBL fold metallo-hydrolase [Thioalkalivibrio sulfidiphilus]	306	306	99%	9e-102	60.80%	WP_012637408.1

MBL fold metallo-hydrolases carry out a variety of biological functions, for example:

Yang, Hanjing, et al. "Evidence that YycJ is a novel 5′–3′ double-stranded DNA exonuclease acting in *Bacillus anthracis* mismatch repair." *DNA repair* 12.5 (2013): 334-346.

Flavodiiron proteins (FDPs) are a family of enzymes endowed with nitric oxide (NO) or oxygen reductase activities, forming the innocuous nitrous oxide (N2O) or water molecules, respectively. FDPs are widespread in the three life kingdoms, and have a modular nature, being each monomer minimally constituted by a metallo-β-lactamase-like domain containing
a catalytic diiron centre, followed by a flavodoxin one, with a flavin mononucleotide.

Folgosa, Filipe, Maria C. Martins, and Miguel Teixeira. "Diversity and complexity of flavodiiron NO/O2 reductases." *FEMS microbiology letters* 365.3 (2018): fnx267.

Peroxiredoxin (sub-family BCP-PrxQ)

>WP_076837529.1:4-149 peroxiredoxin [*Acidihalobacter ferrooxidans*]
IDQAVPDFSAPSTGGDISLHALRGQHVVLYFYPKDNTPGCTTEGTDFRDAHTDFQAAGAVIFGVSRDSLK
SHESFKAKFDFPFELISDTDETLCTLFDVIKMKNMYGKQVRGIERSTFLIDKEGVLRAEWRKVKVPGHCA

Predicted catalytic trio shown in yellow.

Conserved domains on [lcl|Query_8879] View Standard Results

WP_076837529.1:4-149 peroxiredoxin [Acidihalobacter ferrooxidans]

Protein Classification

peroxiredoxin (domain architecture ID 10122458)
peroxiredoxin belonging to the bacterioferritin comigratory protein (BCP) subfamily is a thioredoxin-dependent thiol-specific peroxidase that catalyzes the reduction of hydrogen peroxide and organic hydroperoxides to water and alcohols, respectively

Graphical summary Zoom to residue level show extra options »

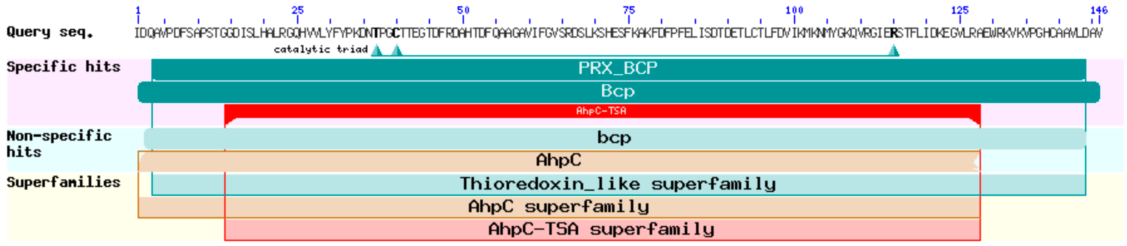

Search for similar domain architectures | Refine search

List of domain hits

Name	Accession	Description	Interval	E-value
[+] PRX_BCP	cd03017	Peroxiredoxin (PRX) family, Bacterioferritin comigratory protein (BCP) subfamily; composed of ...	3-144	5.74e-70
[+] Bcp	COG1225	Peroxiredoxin [Posttranslational modification, protein turnover, chaperones];	1-146	1.24e-68
[+] AhpC-TSA	pfam00578	AhpC/TSA family; This family contains proteins related to alkyl hydroperoxide reductase (AhpC) ...	14-128	9.87e-40
[+] bcp	PRK09437	thioredoxin-dependent thiol peroxidase; Reviewed	2-144	1.18e-28
[+] AhpC	TIGR03137	peroxiredoxin; This peroxiredoxin (AhpC, alkylhydroperoxide reductase subunit C) is one ...	1-128	6.02e-12

Next best BlastP hits after *Acidihalobacter*:

Sequences producing significant alignments Download Manage Columns Show 100

select all *100 sequences selected* GenPept Graphics Distance tree of results Multiple alignment

Description	Max Score	Total Score	Query Cover	E value	Per. Ident	Accession
peroxiredoxin [Paraburkholderia sp.]	226	226	99%	8e-74	74.10%	TAM50272.1
peroxiredoxin [Paraburkholderia sp. PDC91]	226	226	99%	1e-73	74.10%	WP_111487642.1
peroxiredoxin [Paraburkholderia phenazinium]	224	224	99%	9e-73	73.38%	WP_090683940.1
peroxiredoxin [Paraburkholderia phenazinium]	224	224	99%	9e-73	73.38%	WP_074298458.1
peroxiredoxin [Burkholderia dabaoshanensis]	223	223	99%	2e-72	74.10%	WP_102646944.1
peroxiredoxin [Paraburkholderia ginsengiterrae]	223	223	99%	2e-72	72.66%	WP_064269893.1
peroxiredoxin [Paraburkholderia phytofirmans]	223	223	99%	2e-72	72.66%	WP_012432810.1
peroxiredoxin [Paraburkholderia sp. 7MK8-2]	223	223	99%	3e-72	74.10%	WP_121275223.1
peroxiredoxin [Paraburkholderia phenazinium]	223	223	99%	3e-72	72.66%	WP_122154573.1
peroxiredoxin [Paraburkholderia sp. DHOC27]	223	223	99%	3e-72	72.66%	WP_117333767.1
MULTISPECIES: peroxiredoxin [Burkholderiaceae]	223	223	99%	3e-72	72.66%	WP_073426815.1

Some strains of *Paraburkholdaria* and Burkholdaria live at a pH of 4.0–5.0 (e.g. Aizawa, Tomoko, et al. "Burkholderia heleia sp. nov., a nitrogen-fixing bacterium isolated from an aquatic plant, Eleocharis dulcis, that grows in highly acidic swamps in actual acid sulfate soil areas of Vietnam." *International journal of systematic and evolutionary microbiology* 60.5 (2010): 1152-1157 and Xiao, Sen-yang, et al. "Paraburkholderia telluris sp. nov., isolated from

subtropical forest soil." *International journal of systematic and evolutionary microbiology* 69.5 (2019): 1274-1280 and references therein.

Further information about Peroxiredoxin (sub-family BCP-PrxQ):

Jeong, W., Cha, M. K., & Kim, I. H. (2000). Thioredoxin-dependent hydroperoxide peroxidase activity of bacterioferritin comigratory protein (BCP) as a new member of the thiol-specific antioxidant protein (TSA)/Alkyl hydroperoxide peroxidase C (AhpC) family. The Journal of Biological Chemistry, 275(4), 2924-2930.

Kong, W., Shiota, S., Shi, Y., Nakayama, H., & Nakayama, K. (2000). A novel peroxiredoxin of the plant *Sedum lineare* is a homologue of *Escherichia coli* bacterioferritin co-migratory protein (Bcp). The Biochemical Journal, 351(Pt 1), 107-114.

Liao, S., Yang, C., Chin, K., Wang, A. H., & Chou, S. (2009). Insights into the alkyl peroxide reduction pathway of *Xanthomonas campestris* bacterioferritin comigratory protein from the trapped intermediate-ligand complex structures. Journal of Molecular Biology, 390(5), 951-966.

Nelson, K. J., Knutson, S. T., Soito L., Klomsiri C., Poole, L. B., & Fetrow, J. S. (2011). Analysis of the peroxiredoxin family: using active site structure and sequence information for global classification and residue analysis. Proteins, 79(3), 947-964.

Wakita, M., Masuda, S., Motohashi, K., Hisabori, T., Ohta, H., & Takamiya, K. (2007). The significance of type II and PrxQ peroxiredoxins for antioxidative stress response in the purple bacterium *Rhodobacter sphaeroides*. The Journal of Biological Chemistry, 282(38), 27792-27801.
